# Her2 alterations in muscle-invasive bladder cancer: Patient selection beyond protein expression for targeted therapy

**DOI:** 10.1038/srep42713

**Published:** 2017-02-16

**Authors:** Bernhard Kiss, Alexander W. Wyatt, James Douglas, Veronika Skuginna, Fan Mo, Shawn Anderson, Diana Rotzer, Achim Fleischmann, Vera Genitsch, Tetsutaro Hayashi, Maja Neuenschwander, Christine Buerki, Elai Davicioni, Colin Collins, George N. Thalmann, Peter C. Black, Roland Seiler

**Affiliations:** 1 Department of Urology, University of Bern, Bern, Switzerland; 2 Vancouver Prostate Centre, Department of Urologic Sciences, University of British Columbia, Vancouver, Canada; 3 Department of Urology, University Hospital of Southampton, Hampshire SO16 6YD, UK; 4 Institute of Pathology, University of Bern, Bern, Switzerland; 5 Department of Urology, Institute of Biomedical and Health Science, Hiroshima University, Hiroshima, Japan; 6 GenomeDx Biosciences, Inc., Vancouver, British Columbia, Canada

## Abstract

Although the introduction of novel targeted agents has improved patient outcomes in several human cancers, no such advance has been achieved in muscle-invasive bladder cancer (MIBC). However, recent sequencing efforts have begun to dissect the complex genomic landscape of MIBC, revealing distinct molecular subtypes and offering hope for implementation of targeted therapies. Her2 (ERBB2) is one of the most established therapeutic targets in breast and gastric cancer but agents targeting Her2 have not yet demonstrated anti-tumor activity in MIBC. Through an integrated analysis of 127 patients from three centers, we identified alterations of Her2 at the DNA, RNA and protein level, and demonstrate that Her2 relevance as a tumor driver likely may vary even within ERBB2 amplified cases. Importantly, tumors with a luminal molecular subtype have a significantly higher rate of Her2 alterations than those of the basal subtype, suggesting that Her2 activity is also associated with subtype status. Although some of our findings present rare events in bladder cancer, our study suggests that comprehensively assessing Her2 status in the context of tumor molecular subtype may help select MIBC patients most likely to respond to Her2 targeted therapy.

Muscle invasive bladder cancer (MIBC) is a highly aggressive disease, with a 5 year survival rate post-diagnosis of approximately 50%^1^,^2^. Although the implementation of neoadjuvant chemotherapy extended overall patient survival^3^,^4^, prior to the recent advent of immune checkpoint inhibitors, no relevant new therapies have been introduced in the last 3 decades^5^,^6^. This is in stark contrast to several other major cancers^7^,^8^,^9^,^10^,^11^,^12^.

Her2 (gene name: ERBB2) is a member of the epidermal growth factor receptor (EGFR) family, and one of the best-known therapeutic targets in oncology. Her2 can activate intracellular pathways that promote proliferation, survival, mobility and invasiveness of tumor cells and these aggressive oncogenic features translate into reduced survival in patients with Her2-overexpressing breast and gastric cancers^11^,^13^. In these cancers, gene amplification is the primary mechanism for Her2 overexpression and Her2 targeted therapies (e.g. trastuzumab or lapatinib) have become a standard treatment in appropriate tumors^7^,^11^. MIBC has the third highest rate of ERBB2 amplification (after breast and gastric cancer)^14^ and demonstrates frequent Her2 overexpression^15^,^16^. Even so, anti-Her2 treatments in MIBC have not been as encouraging^17^,^18^,^19^,^20^ and despite best practice patient selection by fluorescence *in-situ* hybridization (FISH) and immunohistochemistry (IHC), question whether bladder cancer can respond to Her2 targeted therapy. However, these instruments for patient selection have been developed and shown to be successful in patients with breast or gastric cancers and might not be optimal in those with MIBC.

The identification of tumor molecular subtypes by four separate research groups is one of the most important recent discoveries in MIBC^14^,^21^,^22^,^23^. On a higher level, all represent a division into basal and luminal tumors. Within this framework, each system made specific subclassifications. For example, through RNA profiling of hundreds of MIBC tumors, The Cancer Genome Atlas (TCGA) Research Network identified four distinct clusters that are each associated with specific biological characteristics, pathway activities, and clinical behavior/outcomes^14^. Clusters I and II have predominantly luminal characteristics, express markers of urothelial differentiation such as uroplakins, express the same cytokeratins as the luminal layer of the normal urothelium (KRT18 and KRT20) and exhibit a strong peroxisome proliferator activator receptor (PPAR) pathway activation. Cluster III and IV represent basal tumors, identified by squamous features, expression of cytokeratins (KRT14 and KRT5) and a higher proliferation rate than luminal tumors. These resemble the basal/stem cell compartment of the normal urothelium. In addition, cluster IV tumors show the highest immune infiltration. As a consequence, contemporary biomarker studies must account for the possibility that the baseline characteristics, biological role and significance of genomic alterations may vary between molecular subtypes.

We hypothesized that an integrated approach to Her2 characterization in MIBC may better guide patient prioritization for targeted therapy. Therefore, we assembled a cohort of MIBC patients from three academic centers, identified Her2 alterations at the DNA, RNA and protein level and dissected the relationship of alterations to each other and in the context of the TCGA clusters. We demonstrate that it is necessary to analyze Her2 on all three levels to sufficiently characterize all alterations, and suggest that such comprehensive analysis will provide optimal patient stratification for future Her2-targeted trials.

## Material and Methods

### Patient cohort

We selected a retrospective consecutive cohort of 127 patients from three tertiary centers (Supp Table 1). All patients were diagnosed with muscle-invasive urothelial bladder cancer and clinical staging included computed tomography (CT) scan of the abdomen and pelvis, chest x-ray (or chest CT) and bone scan. All patients received at least 3 cycles of neoadjuvant chemotherapy (NAC) with gemcitabine and cisplatin prior to cystectomy and pelvic lymph node dissection. Patients receiving other chemotherapy regimens or not undergoing cystectomy were excluded. Follow-up varied according to centre, but usually included evaluation at 3 and 6 months postoperatively, every 6-months up to 5 years and yearly thereafter.

### Pathology

Pre-chemotherapy bladder tumor specimens from transurethral resection (TURBT) were used for analysis. All tissue slides from each patient were re-evaluated for this study by two investigators blinded to patient outcome. One haematoxylin and eosin stained section was taken per tissue block and tumor grade was assessed microscopically. In combination with clinical parameters, all tumors were staged according to the seventh International Union Against Cancer (UICC) classification of 2009^24^. A tissue microarray (TMA)^25^ was constructed with two cores per patient from the invasive tumor component.

### Fluorescence *in-situ* hybridization (FISH) and Immunohistochemistry (IHC)

**Figure 1:**
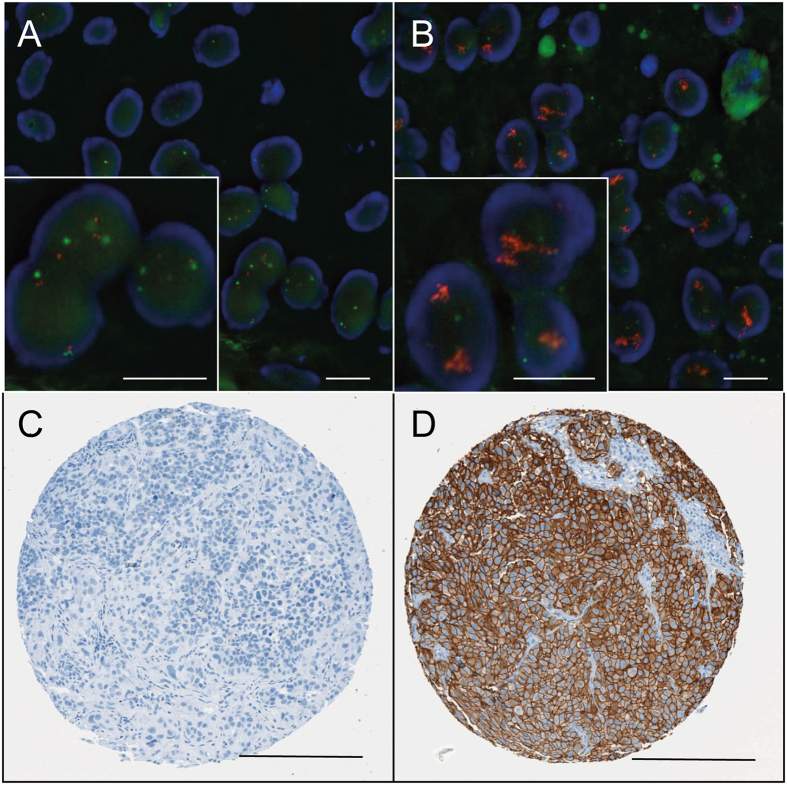
Micrographs of ERBB2 FISH and IHC in bladder cancer samples. Micrographs show representative bladder cancer samples without (**A**) and with (**B**) ERBB2 amplification. ERBB2 yields a red signal while the chromosome 17 centromere is stained with green. Amplification is defined as a gene to centromere ratio ≥2.0 or copy number ≥6.0. Scale bar represents 10 μm. Representative immunohistochemical stains demonstrate Her2 negative (**C**, score 0) and strongly positive bladder cancers (**D**, score 3+). Scale bar represents 200 μm.

Two sections of 3 μm thickness were taken and the FISH reactions were performed according to the FISH Her2 PharmDx (DAKO) protocol. The kit contains a mixture of Texas Red-labeled DNA cosmid clones of the Her2 amplicon and a mixture of fluorescein labeled peptide nucleic acid probes targeting the centromeric region of chromosome 17. The slides were processed according to the manufacturer’s protocol: after deparaffination, sections were immersed in pretreatment solution at 95 °C for 20 minutes followed by pepsin enzymatic digestion (15 minutes) at room temperature. Her2/CEN-17 probe was applied and denatured at 82 °C for 5 minutes. Hybridization was performed overnight at 45 °C. Stringent washes were performed followed by 4,6-diamidino-2-phenylindole staining. Thirty tumor nuclei were evaluated per tissue spot and assigned as negative, equivocal or amplified (Fig. 1A and B and Supp Figure 1A and B) according to the recommendations of the American Society of Clinical Oncology/College of American Pathologists^26^ (Her2/CEP17 ratio <2.0 and Her2 copy number <4.0: negative, ratio <2.0 and Her2 copy number ≥4.0 and <6.0: equivocal, ratio ≥2.0 or copy number ≥6.0: amplified).

The original Hercep Test™ was used for immunohistochemical Her2 protein detection. Her2 expression per tissue spot was classified according to the modified DAKO criteria: negative (0/1+), equivocal (2+) and positive (3+) (Fig. 1C and D and Supp Figure 1C and D) with a cut-off for score 3+ of more than 10% strongly positive cells^26^.

All FISH and IHC staining were performed at the Institute of Pathology, University of Bern, Switzerland. This laboratory is certified, has extensive Her2 IHC and FISH expertise and takes part in external proficiency testing.

### mRNA Expression

Total RNA was extracted from a 1 mm diameter core punch of the tumor with the RNeasy FFPE kit (Qiagen, Valencia, CA), followed by amplification and labeling with the Ovation WTA FFPE system and Encore Biotin Module (both NuGen, San Carlos, CA), respectively. cDNA hybridization to GeneChip Human Exon 1.0 ST oligonucleotide microarrays (Affymetrix, Santa Clara, CA) was performed according to the manufacturer’s recommendations. The Affymetrix Power Tools package was used for quality control^27^ and mRNA expression data was normalized using SCAN^28^. This analysis was performed by GenomeDx Biosciences, a Clinical Laboratory Improvement Amendments (CLIA)-certified laboratory. Array files are available from the National Center for Biotechnology Information’s Gene Expression Omnibus (NCBI–GEO) database (http://www.ncbi.nlm.nih.gov/geo/) under GEO accession code GSE87304.

### Genomic sequencing

DNA was extracted from TURBT specimens from the same cohort; tissue of 96 specimens was available for DNA extraction. Extracted DNA (100 ng) was sheared to 100–400 bp by sonication and was subjected to end-repair, dA-addition, and ligation of indexed IonProton sequencing adaptors. Libraries were hybridization-captured using a pool of >24 000 individually synthesized 50-biotinylated DNA oligonucleotides. In total, 83/96 TURBT specimens passed all QC metrics and had available data on exome DNA sequencing. Synonymous single nucleotide variants (SNV), as well as variants falling in UTRs and introns of ERBB2 were not considered. Non-synonymous (missense) SNVs were called when reported in COSMIC database or with a variant read ≥10 and an allele frequency of ≥10%. All SNVs were validated visually. To determine Copy Number variations (CNV), data analysis was performed using Nexus Copy Number Software, Version 8.0 (BioDiscovery, Hawthorne, CA). Samples were processed using the Nexus NGS functionality (BAM ngCGH) with the FASST2 Segmentation algorithm to make copy number calls (a Circular Binary Segmentation/Hidden Markov Model approach). The significance threshold for segmentation was set at 5 × 10^−6^, also requiring a minimum of 3 probes per segment and a maximum probe spacing of 1000 between adjacent probes before breaking a segment. The logratio thresholds for single copy gain and single copy loss were set at +0.2 and −0.02, respectively. The logratio thresholds for gain of 2 or more copies and for a homozygous loss were set at +0.6 and −1.0, respectively. Tumor sample BAM files were processed with corresponding normal tissue BAM files. Reference reads per CN point (window size) was set at 8000. Probes were normalized to median. The genomic sequencing was performed at laboratory for advanced genomic analysis of the Vancouver Prostate Centre that uses validated and approved experimental protocols.

The number of samples available for each analysis is given in Supp Table 1.

### The TCGA bladder cohort

The TCGA Bladder Urothelial Carcinoma dataset (n = 407) was downloaded from the Broad Institute Firehose Pipeline (http://gdac.broadinstitute.org) on May, 10, 2016. Genomic DNA sequencing with ERBB2 mutation calls (n = 407), CNV (n = 403), gene expression (n = 407), methylation analysis (HM450, n = 407) and reverse phase protein assay (RPPA) data for protein expression (n = 339) were used. For analysis of clinical outcomes only patients without neoadjuvant chemotherapy were selected (n = 397). As above, SNVs that did not alter the amino acid code of ERBB2 were not considered. Missense SNVs were called when reported in COSMIC database or with a variant read ≥10 and an allele frequency of ≥10%.

### Molecular subtyping

In both cohorts (our NAC cohort and TCGA), gene expression data was used to assign tumors to the TCGA clusters. The original “Classification to Nearest Centroids” (ClaNC) model was used^14^.

### Statistical analysis

Statistical analysis was conducted using R Software Package, version 3.1.0. For comparison of non-categorical data between two and more groups logistic regression (lr) was used, respectively. Categorical data were compared using exact Fisher’s test. Pearson correlation was performed to compare the correlation between continuous parameters. Kaplan-Meier plots estimated overall survival (OS) from surgery to the date of death. Patients still living were censored at the date of the last follow-up. Patients were grouped into tertiles of expression for survival analysis stratified according to mRNA expression and RPPA data. Median follow-up was calculated using inverted Kaplan-Meier plots. All tests were with type I error probability of 5%.

### Experimental protocol and ethics

All experiments protocols were approved by the respective institute performing the analysis. The clinical research ethics board of each institution approved this study and all patients consented to analysis of their tumor tissues (Bern, Switzerland KEK-Be 219/2015; Vancouver, Canada H09-01628; Southampton, UK, 10/H0405/99).

## Results

We assembled a cohort of 127 patients with MIBC from three centers. Clinical and pathologic characteristics as well as the number of samples subjected to each mode of analysis (FISH, mRNA expression, IHC) are provided in Supp Table 1. Tissue was derived from the pre-treatment TURBT, and all patients subsequently received NAC.

### Association between ERBB2 gene amplification and expression

**Figure 2:**
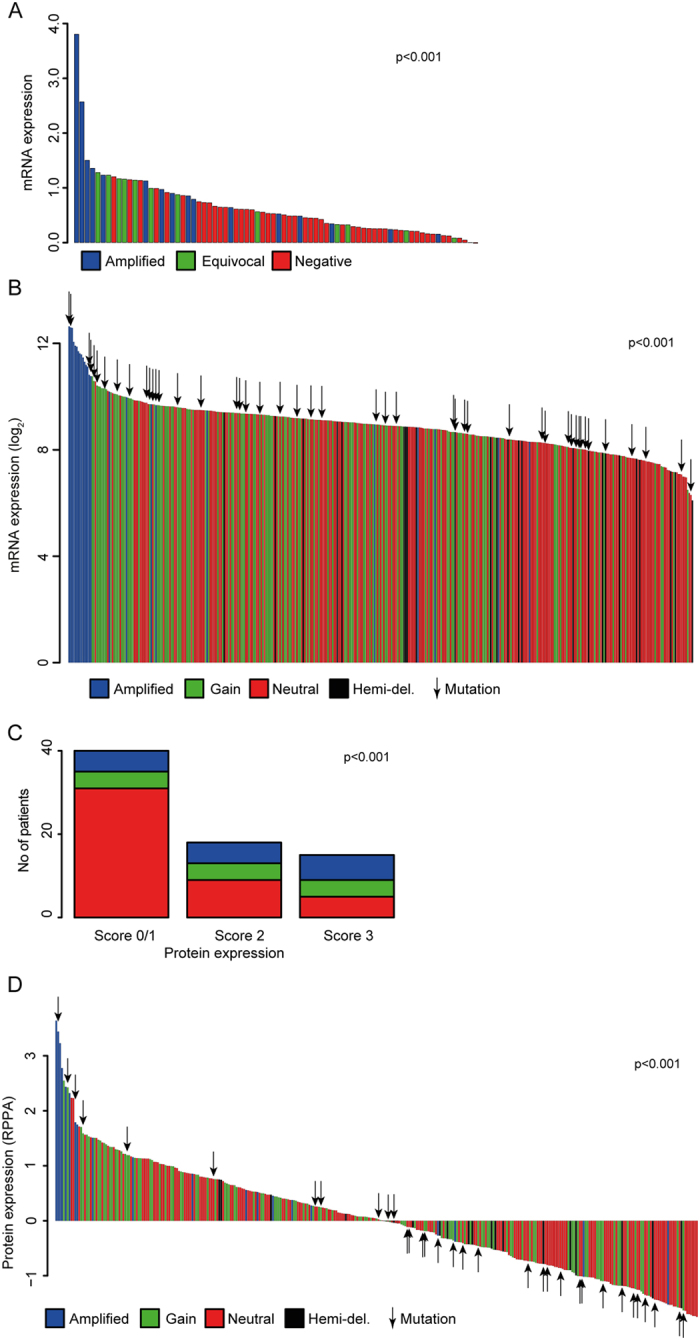
Relationship between ERBB2 gene status and Her2 expression. (**A**) Bar plot demonstrating that ERBB2 amplified MIBC (determined by FISH) was enriched among the samples with high ERBB2 mRNA expression in our NAC cohort (lr, reference level [rl]: ERBB2 amplification status: normal). (**B**) ERBB2 amplified MIBC were similarly enriched among MIBC with a high mRNA expression in the TCGA bladder cohort. Somatic SNVs (indicated by arrows) were evenly distributed between cases with different mRNA expression (lr, mRNA expression vs. CNV, rl: hemizygous deletion). (**C**) Stacked bar plot showing that a significantly higher proportion of tumors with a Her2 protein expression score of 3+ harbored ERBB2 amplification by FISH, than tumors with scores of 0–2 (Fisher’s test). (**D**) Barplot showing Her2 protein expression in the TCGA bladder cohort, indicating significantly higher protein expression in patients with ERBB2 amplification. SNVs are indicated by arrows (lr, protein expression vs. CNV, rl: hemizygous deletion).

In the NAC cohort, FISH revealed that 16/83 tumors harbored ERBB2 amplification, while 24/127 had high Her2 protein expression by IHC (i.e. IHC score = 3). Samples with ERBB2 amplification had higher mRNA (p < 0.001) and protein expression (p < 0.001) (Fig. 2A and C), and mRNA and protein expression were significantly related to each other (p < 0.001) (Supp Figure 2A). However, not all amplified samples had high mRNA and protein expression. Six of 16 amplified samples had mRNA expression in the 1^st^ tertile, and 10/16 had IHC scores of either 1 (n = 5) or 2 (n = 5). Furthermore, 19/67 non-amplified samples had a high mRNA expression (in the 3rd tertile) and 13/83 had an IHC Score 3, suggesting that gene amplification is not the sole driver of high Her2 expression in bladder cancer.

Findings in the TCGA bladder cohort were similar. mRNA and protein expression were significantly higher in amplified samples (p < 0.001, Fig. 2B and D) and correlation between mRNA and protein expression was strong (r: 0.72, p < 0.001, Supp Figure 2B). Not surprisingly, amplified cases had the lowest rate of ERBB2 methylation (p < 0.001, Supp Figure 2C). However, once again a significant proportion of patient tumors (115/403 and 99/339 respectively) without ERBB2 amplification had high (in the 3rd tertile) mRNA and protein expression, and some patients with amplification did not have expression. This may be due to epigenetic factors, since the ERBB2 amplified cases with the lowest mRNA expression had the highest rate of ERBB2 methylation (r: −0.62, p < 0.001, Supp Figure 2D).

### SNVs in ERBB2 and relationship to amplification and expression

**Figure 3:**
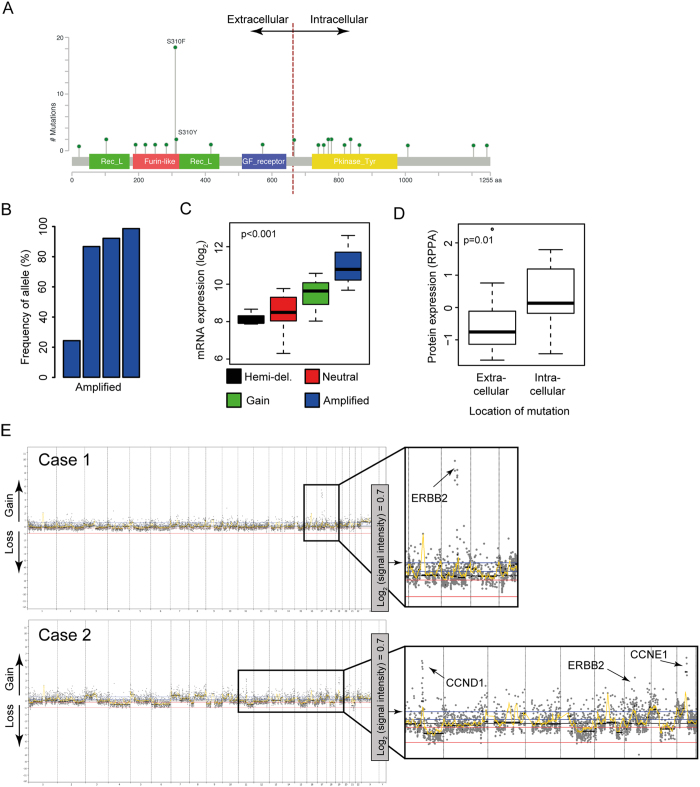
Single nucleotide variants (SNV) in ERBB2 and relationship to amplification and expression. (**A**) Schematic of the ERBB2 coding region showing all somatic missense ERBB2 mutations detected in the TCGA bladder cohort. Twenty of 45 detected SNVs were located in the Furin-like domain. (**B**) Barplot showing the ERBB2 mutant allele frequency in four TCGA cases with both a mutation and concomitant ERBB2 amplification. Note that in 3/4 samples the mutant allele frequency was greater than 85%, suggesting that the SNV is present on all tumor amplicons and occurred prior to gene amplification. (**C**) Boxplot showing that among the 45 TCGA cases with ERBB2 mutations, mRNA expression levels were dependent on copy number status (lr, rl: hemizygous deletion). (**D**) Boxplot demonstrating that cases with Her2 extracellular domain mutations had a significant lower protein expression/detection than samples with intracellular domain mutations (lr, rl: extracellular SNVs). (**E**) Whole genome copy number profiles from two cases in our NAC cohort with ERBB2 amplification. Note that Case 1 (upper panel) appears to harbor isolated ERBB2 amplification, while Case 2 (lower panel) exhibits multiple focal amplifications, including in other well-known oncogenes.

In the TCGA cohort, 45/407 (11%) tumours harbored missense SNVs in ERBB2 (Fig. 3A). Almost half of the mutations (20/45) fell in the Furin-like domain at position 310aa, a well-known mutational hotspot in ERBB2. Interestingly, 4 samples harboured both an SNV and concomitant amplification of ERBB2 (Fig. 3B). Three of these four samples had a mutant allele frequency of >85% which implies that the SNV was present in all alleles and therefore occurred before the amplification of ERBB2. mRNA and protein expression was not significantly different between samples with or without ERBB2 mutations and the expression in samples with ERBB2 mutation appeared to be primarily dependent on copy number variance rather than the location of the mutation (Fig. 3C). Interestingly however, SNVs in the extracellular domain resulted in lower detected protein expression by RPPA compared to SNVs in the intracellular domain (Fig. 3D).

In our own dataset (the NAC cohort), 15/83 (18%) samples had a missense SNV in ERBB2 and 6/15 were in the Furin-like domain. The trend towards lower protein expression/detection in cases with mutation in the extracellular domain was not significant. Importantly, ERBB2 copy number from FISH experiments were significantly related to the copy number estimation (log_2_[ratio]) from exome sequencing, providing robust inter-platform validation (p = 0.03, Supp Figure 2E–G).

We identified four tumors (across both cohorts) with ERBB2 amplification on the background of a relatively ‘quiet’ overall genome copy number profile (one in our NAC: Fig. 3E, upper panel; three in TCGA: Supp Figure 3). Interestingly, three of these ‘exclusively’ ERBB2 amplified tumors had both high Her2 mRNA and protein expression, and were classified in cluster I. The sample with low mRNA and protein expression was classified in cluster III. None of these 4 samples had an ERBB2 SNV. All other samples with ERBB2 amplification also harbored CNV in other well-known oncogenes (e.g. CCND1, CCNE1) (Fig. 3E, lower panel).

### Her2 in relation to TCGA clusters

**Figure 4:**
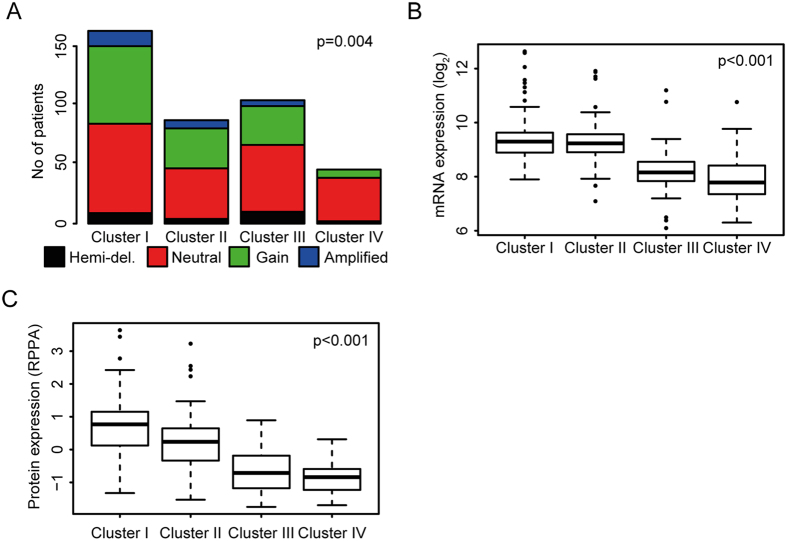
Relationship between Her2 alterations and TCGA clusters. (**A**) In the TCGA bladder cohort, ERBB2 copy number was significantly higher in luminal (cluster I and II) compared to basal tumors (cluster III and IV), but was similar between cluster I and II (Fisher’s test). (**B**) Consistent with our NAC dataset, ERBB2 mRNA expression in the TCGA bladder cohort was higher in cluster I and II than in cluster III and IV (lr, rl: cluster I). (**C**) Her2 protein expression, determined by RPPA was significantly higher in cluster I when compared to all other clusters (lr, rl: cluster I).

In general, Her2 alterations were higher in the luminal TCGA clusters (cluster I and II) compared to the basal clusters (cluster III and IV). In the TCGA cohort, the amplification rate and protein expression were highest in cluster I mRNA expression in cluster I and II was comparable but significantly higher than in cluster III and IV (Fig. 4). Our findings in the NAC cohort were virtually identical (Supp Figure 4). ERBB2 amplification, mRNA and protein expression were highest in cluster I. The amplification rate, mRNA and protein expression were lower in cluster II but still higher than in clusters III and IV.

### Her2 and relation to clinical outcomes

None of the Her2 alterations, including SNVs, were related to pathologic response to NAC when response was defined as no residual muscle invasive disease (<ypT2N0). This was true when considering the entire cohort, and when considering the subtypes separately.

In the entire NAC cohort, Her2 alterations including SNVs were not related to overall survival (Supp Figure 5A,C and E). Similar to findings in the NAC cohort Her2 alterations failed to stratify survival in the entire TCGA bladder cohort (Supp Figure 5B,D and F).

## Discussion

Novel targeted therapies have revolutionized the treatment of many cancers. One of the best examples of this has been targeting of Her2 in overexpressing breast and gastric cancers^7^,^29^,^30^. However, no similar breakthroughs have been achieved in MIBC and inhibition of Her2 has demonstrated scant antitumor activity in clinical trials^17^,^18^,^19^. Targeting Her2 only in MIBC patients with Her2-overexpression and/or ERBB2 amplification was similarly unsuccessful^17^,^18^,^19^,^20^. The reason for this discrepancy between positive Her2 status and lack of antitumor activity is to date unclear. In this study we report that a comprehensive analysis of Her2 alterations at the DNA, RNA and protein levels reveals a complex landscape where only selected tumors have *bona fide* evidence of Her2 driver status. Prior clinical trials investigating Her2-targeted therapies in MIBC have been limited by their use of only FISH and IHC for patient selection^17^,^18^,^19^, and by the absence of Her2 cut-off levels validated specifically for bladder cancer. We posit that our findings may help to enrich future clinical trials investigating Her2-targeted therapies in MIBC with patients most likely to respond.

In general, an increase in ERBB2 copy number resulted in higher mRNA and protein expression in our own and the TCGA cohort. However, ERBB2 amplification was detected without overexpression of Her2 in some cases, while overexpression of Her2 was observed without ERBB2 amplification in others. This suggests that Her2 overexpression is regulated by mechanisms other than gene amplification. Indeed, tumors with ERBB2 amplification but low expression tended to have increased gene methylation, implying an important role for epigenetic control. Even among cases with concomitant overexpression, ERBB2 amplification may be a bystander event in most MIBC. MIBC is characterized by a high rate of genomic alterations^14^, reducing the probability that any individual alteration is a significant and non-redundant oncogenic driver. In rare cases, a combination of ERBB2 amplification with high mRNA and/or protein expression and the absence of other major genomic alterations might be necessary to implicate Her2 as a driver in an individual tumor. In line with data from colorectal cancer^31^, this hypothesis could be verified with patient-derived primary xenografts using Her2 targeted agents. However, this hypothesis will ultimately need testing in clinical trials.

Our findings may suggest that SNVs in the extracellular domain of ERBB2 seem to result in a detection of lower protein expression compared to SNVs in the intracellular domain. This apparent reduced expression may in fact reflect lower affinity of the antibody to the mutated extracellular domain rather than a true alteration of protein expression. Likely, due to the smaller sample size we were not able to validate this finding in our NAC cohort. This finding also might have implications for clinical trials that select patients based on Her2 expression by IHC, where expression may appear artificially low. Furthermore, monoclonal antibodies such as trastuzumab and TDM-1 that target the extracellular domain of Her2 may be rendered ineffective by their inability to bind to the target. Interestingly, somatic mutations in the intracellular tyrosine kinase domain are reported to modulate Her2 signaling, resulting in increased Her2 activation and even resistance to lapatinib in bladder and breast cancer^32^,^33^. In addition, somatic ERBB2 SNVs have been discussed in the context of resistance to trastuzumab^34^. However, none such data exist from bladder cancer. Remarkably, different DNA alterations can occur sequentially, few cases showed SNVs and coincident amplification of ERBB2 with different chronology, a phenomenon that was also noted in a separate cohort with metastasising MIBC^35^. Taken together, beside amplification of the ERBB2-gene, it is likely that further somatic DNA alterations influence success when targeting Her2.

**Figure 5:**
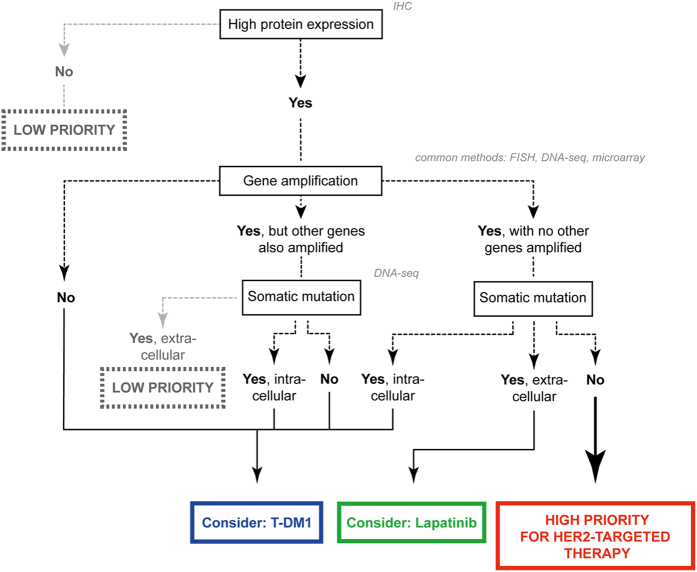
Potential algorithm for molecularly stratifying MIBC patients for Her2 targeted therapy.

Our results suggest that alterations in ERBB2, that are not detectable by ERBB2/HER2 FISH or IHC alone, might have contributed to previously unsuccessful clinical trials of Her2-targeted therapies in MIBC patients. Algorithms for appropriate patient selection for Her2-targeted therapies are well-established and in routine clinical use in breast cancer^26^. However, their direct transfer to MIBC appears to be confounded by the different spectrum of Her2 alterations and the specific context in which they occur (e.g. subtypes, highly mutated and/or unstable genomes). Based upon our study, we propose a potential system for prioritizing patients with MIBC for different Her2-targeted therapies based on specific combinations of Her2 alterations (Fig. 5) that could be explored in clinical trials. In brief, patients with ERBB2 gene amplification but no overexpression of Her2 are likely best treated with alternative therapies. Patients with ERBB2 amplification and Her2 overexpression in association with other major genomic alterations but in the absence of SNVs in the extracellular domain may benefit from TDM-1, the antibody drug-conjugate that requires Her2 overexpression for delivery of the cytotoxic payload, but is independent of Her2 pathway activity^36^. Finally, patients with ERBB2 gene amplification and protein overexpression in the absence of ERBB2 SNVs and other major genomic alterations may benefit most from trastuzumab or lapatinib (or other Her2-targeting tyrosine kinase inhibitors). As described above, mutations in either the extracellular or intracellular domain may have implications for trastuzumab or tyrosine kinase inhibitors, respectively. We are aware that this blueprint must be clinically qualified, and remains speculative in the meantime.

Recently elucidated molecular subtypes of MIBC based on gene expression are providing a framework for dissecting bladder cancer biology 14,21,22,23. Our results reinforce the need to consider potential biomarkers in the context of molecular subtypes. We observed that ERBB2 amplification, mRNA expression and protein expression were all significantly higher in tumors with luminal characteristics (cluster I and II). It is entirely plausible that genuine biological differences between basal and luminal MIBC influenced the results of recent clinical trials^20^. For example, lapatinib was considered particularly desirable via its dual inhibition of Her1 and Her2. On this basis, Her1 and/or Her2 positive patients were selected by IHC for enrollment in this trial^20^. Even excluding the likelihood that Her1 and Her2 overexpressing MIBC are biologically distinct entities^21^,^22^,^23^, the overall survival differences between patients with basal (enriched with Her1 positive) and luminal (enriched with Her2 positive) MIBC would probably confound trial outcome interpretation. Underscoring this hypothesis is our result showing that despite their established and potent oncogenic effect, Her2 alterations were not associated with poor overall survival.

Our study is not without limitations that are mainly due to its retrospective character. The focus of this manuscript was not to investigate the prognostic impact of Her2 in MIBC but to elucidate the intricacies of Her2 alterations at the DNA, RNA and protein levels, and highlight the significance of molecular subtypes in this context. The amplification rate of 19% in our NAC cohort is high compared to previous data including to our own experience of ERBB2 amplification in MIBC^37^. However, we were able validate our FISH data by genomic DNA sequencing. In addition, the threshold for ERBB2 amplification determined by FISH has been modified^26^. According to the historical threshold, the amplification rate in our NAC cohort would be 12%, which is consistent with previous findings in MIBC.

Our study of ERBB2/Her2 is exemplary for the type of analysis that may be necessary also for other targets in MIBC. FGFR3 alterations, for example vary according to protein expression, mutation, gene fusion and copy number, and it is not clear which is most relevant for success of FGFR3-targeted therapy. This is in apparent contrast to the success of targeted therapy in breast and gastric cancer where exclusive evaluation of Her2 protein expression via IHC (at least in patients with score 3) is sufficient to prioritize patients for Her2 targeted therapy. It is plausibly explained in these cancers by high concordance between gene status determined by FISH and protein expression (87.5% in gastric cancer^38^ and breast cancer 87.3%^39^). Contrarily, this concordance is lower in MIBC, which also has a higher rate of alterations across the entire genome. This latter characteristic may make FISH or IHC alone insufficient for selection of MIBC patients for Her2 targeted therapy.

## Conclusions

Assessment of Her2 alterations at the DNA, RNA and protein level provides a much more comprehensive insight into Her2 relevance as a driver gene and a therapeutic target in MIBC, than through FISH or IHC alone. The complex molecular landscape of Her2 alteration has probably confounded previous clinical trials of Her2-targeted therapies, and improved patient selection accounting for multi-modal Her2 status and tumor molecular subtype will be essential for future clinical trial design.

## Additional Information

**How to cite this article:** Kiss, B. *et al*. Her2 alterations in muscle-invasive bladder cancer: Patient selection beyond protein expression for targeted therapy. *Sci. Rep.* **7**, 42713; doi: 10.1038/srep42713 (2017).

**Publisher's note:** Springer Nature remains neutral with regard to jurisdictional claims in published maps and institutional affiliations.

## Supplementary Material

Supplementary Text
