# Supplementary material for: Her2 alterations in muscle-invasive bladder cancer: Patient selection beyond protein expression for targeted therapy
Source: Sci Rep. 2017 Feb 16;7:42713. doi: 10.1038/srep42713 (PMC5311866; doi:10.1038/srep42713)
Supplement: Supplementary Text [file srep42713-s1.doc]

**SUPPLEMENTARY FILES**

**Her2 alterations in muscle-invasive bladder cancer: Patient selection beyond protein expression for targeted therapy**

Bernhard Kiss, Alexander W. Wyatt, James Douglas, Veronika Skuginna, Fan Mo, Shawn Anderson, Diana Rotzer, Achim Fleischmann, Vera Genitsch, Tetsutaro Hayashi, Maja Neuenschwander, Christine Bürki, Elai Davicioni, Colin Collins, George N. Thalmann, Peter C. Black, Roland Seiler

**Supplementary Table 1** Clinical and pathological data of the assembled NAC cohort.

| **Patient demographis (n=127)** |  |
| --- | --- |
| Age (median, range) at surgery (years) | 63 (35-81) |
| Gender (female/male) | 36/91 |
| Follow-up (median, range) (years) | 3.5 (0.1-12.4) |
| **Cystectomy and lymphadenectomy data** |  |
| Tumor stage (n) |  |
| ypT0/1 (%) | 50 (39) |
| ypT2 (%) | 29 (23) |
| ypT3/4 (%) | 48 (38) |
| Lymph node stage (n) |  |
| ypN0 (%) | 95 (75) |
| ypN1-3 (%) | 32 (25) |
| **Samples available for analysis** |  |
| Whole exome sequencing | 83/127 |
| FISH | 83/127 |
| mRNA expression | 127/127 |
| IHC | 115/127 |

**Supplementary Table 2**. Genes with known frequent alterations in bladder cancer that are included in analysis in Supp. Fig. 3.

| **Gene** | **Relevance** |
| --- | --- |
| AKT1 | PI3K pathway |
| APC | WNT signaling |
| ARID1A | SWI/SNG chromatin |
| ASXL2 | Polycomb |
| ATM | DNA damage repair |
| BAP1 | DNA damage repair |
| BRAF | Oncogene |
| CCND1 | CDK |
| CCNE1 | CDK pathway |
| CDKN1A | CDK inhibitor |
| CDKN2A | CDK inhibitor |
| CREBBP | cAMP |
| CTNNB1 | WNT signaling |
| E2F3 | RB1 related |
| EGFR | Oncogene |
| ELF3 | ETS |
| EP300 | Histone acetyltransferase |
| ERBB2 | PI3K pathway |
| ERBB3 | RTK |
| ERCC2 | DNA damage repair |
| FANCC | DNA damage repair |
| FBXW7 | F-box family |
| FGFR1 | FGF pathway |
| FGFR3 | FGF pathway |
| FOXA1 | forkhead tf |
| FOXQ1 | forkhead tf |
| HRAS | RAS oncogene |
| KDM6A | Histone demethylase |
| KLF5 | Kruppel-like tf |
| KMT2D | Histone methyltransferase (MLL2) |
| KRAS | RAS oncogene |
| MDM2 | TP53 ubiq |
| MET | Oncogene |
| MYC | Oncogene |
| NF1 | RAS pathway |
| NFE2L2 | stress |
| NFE2L3 | stress |
| NOTCH1 | Notch |
| NOTCH2 | Notch |
| NOTCH3 | Notch |
| NRAS | RAS oncogene |
| PIK3CA | PI3K pathway |
| PIK3R1 | PI3K pathway |
| PPARG | Retinoic acid related |
| PTCH1 | SHH pathway |
| PTEN | PI3K pathway |
| RB1 | tumor suppressor |
| RHOA | Varied |
| RHOB | Varied |
| RUNX1 | Runt-domain |
| RUNX3 | Runt-domain |
| RXRA | Retinoic acid |
| STAG2 | DNA replication |
| TERT | Cancer associated |
| TP53 | tumor suppressor |
| TSC1 | mTOR pathway |
| TSC2 | mTOR pathway |
| TXNIP | Thioredoxin-interacting |
| ZBTB7B | Immune related |
| ZFP36L1 | RNA-binding |

**Supplementary Table 3** SNVs, FISH, Protein expression data and subtype membership of the NAC cohort.

| celfile.name | FISH | SNV | SNV.location | mRNA | IHC | TCGA.cluster |
| --- | --- | --- | --- | --- | --- | --- |
| GDX-13207-AI426-HuEx-1_0-st-v2-01-1 (NAC001).CEL | NA | WT | NA | 0.291945025 | Score 0/1 | ClusterII |
| GDX-9990-gbx-NAC002-@52107700960933012617425462083653-20150605.CEL | NA | WT | NA | 0.688984631 | Score 3 | ClusterI |
| GDX-9991-gbx-NAC003-@52107700960933012617425462083673-20150605.CEL | NA | WT | NA | 0.397095239 | Score 0/1 | ClusterI |
| GDX-13212-AI431-HuEx-1_0-st-v2-01-1 (NAC004).CEL | NA | MUT | 450 | 0.059303561 | Score 0/1 | ClusterIV |
| GDX-13203-AI422-HuEx-1_0-st-v2-01-1 (NAC005).CEL | Equivocal | WT | NA | 1.159414853 | Score 2 | ClusterI |
| GDX-13980-AI717-HuEx-1_0-st-v2-01-1 (NAC006).CEL | NA | NA | NA | -0.030199815 | NA | NA |
| GDX-13885-AI666-HuEx-1_0-st-v2-01-1 (NAC007).CEL | NA | WT | NA | 0.272659116 | Score 0/1 | ClusterIII |
| GDX-13787-AI591-HuEx-1_0-st-v2-01-1 (NAC008).CEL | NA | WT | NA | 0.121786441 | Score 0/1 | ClusterIII |
| GDX-13643-AI540-HuEx-1_0-st-v2-01-1 (NAC009).CEL | NA | WT | NA | 0.132906355 | Score 0/1 | NA |
| GDX-13766-AI570-HuEx-1_0-st-v2-01-1 (NAC010).CEL | NA | WT | NA | 0.408203791 | Score 3 | ClusterII |
| GDX-13979-AI716-HuEx-1_0-st-v2-01-1 (NAC011).CEL | Normal | WT | NA | 0.726824218 | Score 0/1 | ClusterI |
| GDX-13953-AI690-HuEx-1_0-st-v2-01-1 (NAC012).CEL | NA | WT | NA | 0.321775827 | Score 0/1 | ClusterI |
| GDX-13187-AI409-HuEx-1_0-st-v2-01-1 (NAC013).CEL | NA | WT | NA | 0.814660987 | Score 3 | ClusterI |
| GDX-13628-AI525-HuEx-1_0-st-v2-01-1 (NAC014).CEL | NA | WT | NA | 0.136663311 | Score 0/1 | NA |
| GDX-13618-AI515-HuEx-1_0-st-v2-01-1 (NAC015).CEL | NA | WT | NA | 0.051719123 | Score 0/1 | NA |
| GDX-13775-AI579-HuEx-1_0-st-v2-01-1 (NAC016).CEL | NA | WT | NA | 0.153200391 | Score 0/1 | NA |
| GDX-13871-AI652-HuEx-1_0-st-v2-01-1 (NAC017).CEL | Amplified | WT | NA | 3.80600256 | Score 3 | ClusterIII |
| GDX-13776-AI580-HuEx-1_0-st-v2-01-1 (NAC018).CEL | Amplified | WT | NA | 0.239849592 | Score 3 | ClusterIV |
| GDX-13647-AI544-HuEx-1_0-st-v2-01-1 (NAC019).CEL | Normal | MUT | 310 | 0.665371998 | Score 0/1 | ClusterI |
| GDX-13646-AI543-HuEx-1_0-st-v2-01-1 (NAC020).CEL | NA | WT | NA | 0.25717324 | Score 0/1 | ClusterIV |
| GDX-13620-AI517-HuEx-1_0-st-v2-01-1 (NAC021).CEL | NA | WT | NA | 0.568840369 | Score 2 | ClusterI |
| GDX-13872-AI653-HuEx-1_0-st-v2-01-1 (NAC022).CEL | Normal | WT | NA | 0.205859697 | Score 0/1 | ClusterII |
| GDX-13270-AI308-HuEx-1_0-st-v2-01-1 (NAC023).CEL | Normal | WT | NA | 0.988791299 | Score 3 | ClusterII |
| GDX-13188-AI410-HuEx-1_0-st-v2-01-1 (NAC024).CEL | NA | MUT | 310 | 0.446607414 | Score 0/1 | ClusterII |
| GDX-13192-AI411-HuEx-1_0-st-v2-01-1 (NAC025).CEL | NA | NA | NA | 0.852557711 | Score 0/1 | ClusterI |
| GDX-13782-AI586-HuEx-1_0-st-v2-01-1 (NAC027).CEL | Normal | WT | NA | 0.225026174 | Score 0/1 | ClusterIV |
| GDX-13971-AI708-HuEx-1_0-st-v2-01-1 (NAC028).CEL | NA | WT | NA | 0.953277386 | Score 3 | ClusterI |
| GDX-13856-AI635-HuEx-1_0-st-v2-01-1 (NAC029).CEL | Normal | WT | NA | 0.253375231 | Score 0/1 | ClusterIII |
| GDX-13206-AI425-HuEx-1_0-st-v2-01-1 (NAC030).CEL | NA | WT | NA | 0.537064943 | Score 0/1 | ClusterII |
| GDX-13759-AI563-HuEx-1_0-st-v2-01-1 (NAC032).CEL | Normal | WT | NA | 0.605741606 | Score 2 | ClusterII |
| GDX-13275-AI313-HuEx-1_0-st-v2-01-1 (NAC033).CEL | Normal | WT | NA | 0.60880031 | Score 0/1 | ClusterIV |
| GDX-13850-AI629-HuEx-1_0-st-v2-01-1 (NAC034).CEL | NA | NA | NA | 0.976476518 | Score 0/1 | ClusterI |
| GDX-13624-AI521-HuEx-1_0-st-v2-01-1 (NAC035).CEL | NA | MUT | 1000 | 0.278989078 | Score 3 | ClusterII |
| GDX-13866-AI645-HuEx-1_0-st-v2-01-1 (NAC036).CEL | Normal | WT | NA | 0.083043196 | Score 0/1 | ClusterIV |
| GDX-13855-AI634-HuEx-1_0-st-v2-01-1 (NAC037).CEL | NA | MUT | 767 | -0.039108967 | Score 0/1 | NA |
| GDX-9993-gbx-NAC038-@52107700960935012617425462083773-20150605.CEL | NA | WT | NA | 1.349462724 | Score 0/1 | ClusterII |
| GDX-13879-AI660-HuEx-1_0-st-v2-01-1 (NAC039).CEL | Normal | NA | NA | 0.529160645 | Score 3 | ClusterII |
| GDX-13621-AI518-HuEx-1_0-st-v2-01-1 (NAC040).CEL | Normal | WT | NA | 0.235078846 | Score 0/1 | ClusterIII |
| GDX-13767-AI571-HuEx-1_0-st-v2-01-1 (NAC041).CEL | Amplified | WT | NA | 1.503148098 | Score 3 | ClusterII |
| GDX-13851-AI630-HuEx-1_0-st-v2-01-1 (NAC042).CEL | Equivocal | WT | NA | 0.876221797 | Score 2 | ClusterI |
| GDX-13770-AI574-HuEx-1_0-st-v2-01-1 (NAC043).CEL | Normal | WT | NA | 0.44576725 | Score 2 | ClusterIII |
| GDX-13957-AI694-HuEx-1_0-st-v2-01-1 (NAC044).CEL | Normal | WT | NA | 0.858856775 | Score 2 | ClusterI |
| GDX-13803-AI607-HuEx-1_0-st-v2-01-1 (NAC046).CEL | Equivocal | WT | NA | 1.167330464 | Score 3 | ClusterI |
| GDX-13976-AI713-HuEx-1_0-st-v2-01-1 (NAC047).CEL | Equivocal | MUT | 769 | 1.27990025 | Score 2 | ClusterI |
| GDX-13658-AI555-HuEx-1_0-st-v2-01-1 (NAC048).CEL | Normal | WT | NA | 0.448491325 | Score 2 | ClusterII |
| GDX-13642-AI539-HuEx-1_0-st-v2-01-1 (NAC049).CEL | Normal | MUT | 842 | 0.747464773 | Score 2 | ClusterII |
| GDX-13877-AI658-HuEx-1_0-st-v2-01-1 (NAC050).CEL | Amplified | WT | NA | 0.791786665 | Score 3 | ClusterI |
| GDX-13218-AI437-HuEx-1_0-st-v2-01-1 (NAC051).CEL | Amplified | NA | NA | 0.853905179 | Score 0/1 | ClusterI |
| GDX-13854-AI633-HuEx-1_0-st-v2-01-1 (NAC052).CEL | Normal | WT | NA | 0.487173438 | Score 0/1 | ClusterI |
| GDX-13758-AI562-HuEx-1_0-st-v2-01-1 (NAC053).CEL | Normal | WT | NA | 0.326466178 | Score 0/1 | ClusterIV |
| GDX-13998-AI736-HuEx-1_0-st-v2-01-1 (NAC054).CEL | Normal | WT | NA | 0.179537401 | Score 0/1 | NA |
| GDX-9994-gbx-NAC055-@52107700960936012617425462083840-20150605.CEL | Equivocal | WT | NA | 1.231678501 | Score 3 | ClusterI |
| GDX-13994-AI732-HuEx-1_0-st-v2-01-1 (NAC056).CEL | Equivocal | WT | NA | 0.217737596 | Score 3 | ClusterII |
| GDX-13780-AI584-HuEx-1_0-st-v2-01-1 (NAC057).CEL | Amplified | WT | NA | 0.90021089 | Score 2 | ClusterI |
| GDX-13846-AI625-HuEx-1_0-st-v2-01-1 (NAC058).CEL | Normal | MUT | 1079 | 1.203406301 | Score 3 | ClusterI |
| GDX-13201-AI420-HuEx-1_0-st-v2-01-1 (NAC091).CEL | NA | NA | NA | 0.112901553 | Score 0/1 | ClusterIV |
| GDX-13956-AI693-HuEx-1_0-st-v2-01-1 (NAC092).CEL | Normal | WT | NA | 0.507696146 | Score 0/1 | ClusterIII |
| GDX-13991-AI728-HuEx-1_0-st-v2-01-1 (NAC093).CEL | Amplified | NA | NA | 1.358268409 | Score 0/1 | ClusterII |
| GDX-13890-AI671-HuEx-1_0-st-v2-01-1 (NAC094).CEL | NA | WT | NA | -0.023922705 | Score 0/1 | ClusterII |
| GDX-13987-AI724-HuEx-1_0-st-v2-01-1 (NAC095).CEL | Normal | WT | NA | 1.148193382 | Score 3 | ClusterI |
| GDX-13970-AI707-HuEx-1_0-st-v2-01-1 (NAC096).CEL | NA | WT | NA | 0.69210549 | Score 0/1 | ClusterIII |
| GDX-13633-AI530-HuEx-1_0-st-v2-01-1 (NAC097).CEL | Equivocal | WT | NA | 0.085352727 | Score 0/1 | ClusterIII |
| GDX-13794-AI598-HuEx-1_0-st-v2-01-1 (NAC098).CEL | Normal | MUT | 500 | 0.612027878 | Score 2 | ClusterII |
| GDX-13888-AI669-HuEx-1_0-st-v2-01-1 (NAC099).CEL | Normal | WT | NA | 0.55664491 | Score 2 | ClusterII |
| GDX-13652-AI549-HuEx-1_0-st-v2-01-1 (NAC100).CEL | NA | WT | NA | 0.015696706 | Score 0/1 | ClusterIV |
| GDX-13655-AI552-HuEx-1_0-st-v2-01-1 (NAC101).CEL | NA | NA | NA | 0.096339583 | Score 0/1 | ClusterIII |
| GDX-13983-AI720-HuEx-1_0-st-v2-01-1 (NAC102).CEL | Amplified | NA | NA | 2.57070924 | Score 3 | ClusterII |
| GDX-13959-AI696-HuEx-1_0-st-v2-01-1 (NAC103).CEL | NA | MUT | 310 | 0.121145932 | Score 0/1 | ClusterIII |
| GDX-13788-AI592-HuEx-1_0-st-v2-01-1 (NAC105).CEL | Normal | WT | NA | -0.011986673 | Score 0/1 | NA |
| GDX-13760-AI564-HuEx-1_0-st-v2-01-1 (NAC106).CEL | Normal | WT | NA | 0.351140497 | Score 0/1 | ClusterIII |
| GDX-13793-AI597-HuEx-1_0-st-v2-01-1 (NAC107).CEL | Normal | MUT | 697 | 0.293257981 | Score 0/1 | ClusterIII |
| GDX-13986-AI723-HuEx-1_0-st-v2-01-1 (NAC108-2).CEL | Equivocal | NA | NA | 0.325140782 | Score 0/1 | ClusterII |
| GDX-13651-AI548-HuEx-1_0-st-v2-01-1 (NAC109).CEL | Equivocal | MUT | 769 | 0.331381934 | Score 0/1 | ClusterIII |
| GDX-13195-AI414-HuEx-1_0-st-v2-01-1 (NAC110).CEL | Normal | WT | NA | 0.283224752 | Score 0/1 | ClusterIII |
| GDX-13792-AI596-HuEx-1_0-st-v2-01-1 (NAC111).CEL | NA | NA | NA | 1.333321606 | Score 0/1 | ClusterI |
| GDX-13891-AI672-HuEx-1_0-st-v2-01-1 (NAC112).CEL | NA | NA | NA | 0.137204101 | Score 0/1 | ClusterIII |
| GDX-13785-AI589-HuEx-1_0-st-v2-01-1 (NAC113).CEL | Equivocal | WT | NA | 1.140345588 | Score 3 | ClusterII |
| GDX-13641-AI538-HuEx-1_0-st-v2-01-1 (NAC114).CEL | NA | NA | NA | 0.234707951 | Score 3 | ClusterI |
| GDX-6851-BER001.CEL | Amplified | NA | NA | 1.231750706 | Score 2 | ClusterI |
| GDX-6236-BER004.CEL | Normal | NA | NA | 0.123135094 | Score 0/1 | ClusterIV |
| GDX-6830-BER007.CEL | Normal | NA | NA | 0.645829958 | Score 0/1 | ClusterI |
| GDX-6183-BER009.CEL | Amplified | NA | NA | 0.484449965 | Score 2 | ClusterI |
| GDX-6212-BER011.CEL | Amplified | NA | NA | 0.640274505 | Score 0/1 | NA |
| GDX-6181-BER012.CEL | Amplified | WT | NA | 0.971645768 | Score 2 | ClusterI |
| GDX-6843-BER015.CEL | Normal | WT | NA | 0.914575127 | Score 2 | ClusterI |
| GDX-6596-BER017.CEL | NA | NA | NA | 0.435140529 | Score 2 | NA |
| GDX-6595-BER020.CEL | NA | NA | NA | 0.132885192 | Score 0/1 | NA |
| GDX-6566-BER022.CEL | Amplified | NA | NA | 0.153538111 | Score 0/1 | ClusterIII |
| GDX-6592-BER025.CEL | Normal | WT | NA | 0.423666204 | Score 0/1 | NA |
| GDX-6569-BER027.CEL | Normal | NA | NA | 0.156691176 | NA | ClusterII |
| GDX-6170-BER029.CEL | Normal | WT | NA | 0.732949929 | Score 0/1 | ClusterI |
| GDX-6582-BER030.CEL | Normal | WT | NA | 0.644384216 | Score 0/1 | ClusterII |
| GDX-6571-BER033.CEL | Normal | WT | NA | 1.135074377 | Score 2 | ClusterII |
| GDX-6239-BER035.CEL | NA | NA | NA | 0.514169552 | Score 3 | ClusterI |
| GDX-6840-BER036.CEL | Amplified | NA | NA | 0.343437164 | Score 0/1 | ClusterII |
| GDX-6854-BER038.CEL | Normal | NA | NA | 0.53298843 | Score 0/1 | ClusterII |
| GDX-6237-BER045.CEL | NA | NA | NA | 0.992646895 | NA | ClusterI |
| GDX-6223-BER049.CEL | Normal | NA | NA | 0.161511204 | Score 0/1 | ClusterIII |
| GDX-6573-BER050.CEL | Amplified | MUT | 310 | 0.525606233 | Score 2 | ClusterI |
| GDX-6214-BER053.CEL | NA | NA | NA | 0.404065468 | Score 0/1 | ClusterIII |
| GDX-6219-BER055.CEL | Normal | NA | NA | 0.254749728 | Score 0/1 | ClusterIII |
| GDX-6575-BER058.CEL | Equivocal | NA | NA | 0.990601282 | Score 2 | ClusterI |
| GDX-6837-BER059-2.CEL | NA | NA | NA | 0.146443179 | Score 0/1 | ClusterIII |
| GDX-6209-BER061.CEL | Normal | NA | NA | 0.599902024 | NA | ClusterI |
| GDX-6241-BER062.CEL | Normal | NA | NA | 0.003840019 | NA | ClusterII |
| GDX-6589-BER063.CEL | Equivocal | WT | NA | 0.564685276 | Score 0/1 | NA |
| GDX-6844-BER066.CEL | Normal | NA | NA | 0.487731179 | Score 0/1 | ClusterII |
| GDX-6588-BER067.CEL | NA | WT | NA | 0.527518927 | Score 3 | ClusterII |
| GDX-6175-BER068.CEL | NA | NA | NA | 0.757294129 | Score 2 | ClusterI |
| GDX-6178-BER069.CEL | NA | NA | NA | 0.641343978 | Score 2 | ClusterII |
| GDX-6587-BER072.CEL | Normal | MUT | 310 | 0.258229225 | Score 0/1 | NA |
| GDX-6577-BER074.CEL | Normal | NA | NA | 0.251919366 | Score 0/1 | ClusterI |
| GDX-6174-BER076.CEL | NA | NA | NA | 0.092208512 | NA | ClusterIII |
| GDX-6842-BER079.CEL | NA | NA | NA | 0.659779283 | Score 2 | ClusterII |
| GDX-6216-BER081.CEL | NA | NA | NA | 0.141915727 | NA | ClusterII |
| GDX-6826-BER082.CEL | Normal | WT | NA | 0.208146612 | Score 0/1 | ClusterIII |
| GDX-6221-BER086.CEL | Normal | NA | NA | 0.455466015 | Score 3 | ClusterI |
| GDX-6590-BER094.CEL | NA | MUT | 310 | 0.333641255 | Score 2 | ClusterII |
| GDX-6579-BER096.CEL | Amplified | NA | NA | 1.126789285 | Score 3 | NA |
| GDX-6228-BER098.CEL | Normal | NA | NA | 0.128719478 | Score 0/1 | ClusterIV |
| GDX-6594-BER101.CEL | Normal | NA | NA | 0.264073888 | Score 0/1 | NA |
| GDX-6828-BER103.CEL | Normal | WT | NA | 0.046079691 | Score 0/1 | ClusterIV |
| GDX-6188-BER104.CEL | NA | WT | NA | 0.151747535 | NA | ClusterIII |
| GDX-6180-BER106.CEL | NA | NA | NA | 0.819924037 | NA | ClusterII |
| GDX-6206-BER108.CEL | NA | WT | NA | 0.327135833 | NA | NA |
| GDX-6242-BER114.CEL | NA | NA | NA | 0.316803026 | NA | ClusterIII |
| GDX-6583-BER118.CEL | NA | NA | NA | 0.405567923 | NA | ClusterIV |

**Supplementary Table 4** Subtype membership of the 407 tumors in TCGA.

| Sample_ID | tcga_cluster |
| --- | --- |
| TCGA-2F-A9KP-01A-11R-A38B-07 | Cluster_I |
| TCGA-2F-A9KQ-01A-11R-A38B-07 | Cluster_I |
| TCGA-2F-A9KR-01A-11R-A38B-07 | Cluster_I |
| TCGA-2F-A9KT-01A-11R-A38B-07 | Cluster_I |
| TCGA-2F-A9KW-01A-11R-A38B-07 | Cluster_I |
| TCGA-4Z-AA7M-01A-11R-A39I-07 | Cluster_I |
| TCGA-4Z-AA7N-01A-11R-A39I-07 | Cluster_IV |
| TCGA-4Z-AA7O-01A-31R-A39I-07 | Cluster_I |
| TCGA-4Z-AA7Q-01A-11R-A39I-07 | Cluster_III |
| TCGA-4Z-AA7R-01A-11R-A39I-07 | Cluster_I |
| TCGA-4Z-AA7S-01A-11R-A39I-07 | Cluster_I |
| TCGA-4Z-AA7W-01A-11R-A39I-07 | Cluster_IV |
| TCGA-4Z-AA7Y-01A-11R-A39I-07 | Cluster_I |
| TCGA-4Z-AA80-01A-11R-A39I-07 | Cluster_I |
| TCGA-4Z-AA81-01A-11R-A39I-07 | Cluster_I |
| TCGA-4Z-AA82-01A-11R-A39I-07 | Cluster_III |
| TCGA-4Z-AA83-01A-11R-A39I-07 | Cluster_I |
| TCGA-4Z-AA84-01A-11R-A39I-07 | Cluster_III |
| TCGA-4Z-AA86-01A-11R-A39I-07 | Cluster_III |
| TCGA-4Z-AA87-01A-11R-A39I-07 | Cluster_I |
| TCGA-4Z-AA89-01A-11R-A39I-07 | Cluster_I |
| TCGA-5N-A9KI-01A-31R-A42T-07 | Cluster_II |
| TCGA-5N-A9KM-01A-11R-A42T-07 | Cluster_II |
| TCGA-BL-A0C8-01A-11R-A10U-07 | Cluster_I |
| TCGA-BL-A13I-01A-11R-A13Y-07 | Cluster_IV |
| TCGA-BL-A13J-01A-11R-A10U-07 | Cluster_II |
| TCGA-BL-A3JM-01A-12R-A21D-07 | Cluster_III |
| TCGA-BL-A5ZZ-01A-31R-A30C-07 | Cluster_IV |
| TCGA-BT-A0S7-01A-11R-A10U-07 | Cluster_III |
| TCGA-BT-A0YX-01A-11R-A10U-07 | Cluster_III |
| TCGA-BT-A20J-01A-11R-A14Y-07 | Cluster_IV |
| TCGA-BT-A20N-01A-11R-A14Y-07 | Cluster_I |
| TCGA-BT-A20O-01A-21R-A14Y-07 | Cluster_IV |
| TCGA-BT-A20P-01A-11R-A14Y-07 | Cluster_I |
| TCGA-BT-A20Q-01A-11R-A14Y-07 | Cluster_II |
| TCGA-BT-A20R-01A-12R-A16R-07 | Cluster_II |
| TCGA-BT-A20T-01A-11R-A14Y-07 | Cluster_II |
| TCGA-BT-A20U-01A-11R-A14Y-07 | Cluster_III |
| TCGA-BT-A20V-01A-11R-A14Y-07 | Cluster_III |
| TCGA-BT-A20W-01A-21R-A14Y-07 | Cluster_I |
| TCGA-BT-A20X-01A-11R-A16R-07 | Cluster_III |
| TCGA-BT-A2LA-01A-11R-A18C-07 | Cluster_III |
| TCGA-BT-A2LB-01A-11R-A18C-07 | Cluster_II |
| TCGA-BT-A2LD-01A-12R-A20F-07 | Cluster_III |
| TCGA-BT-A3PH-01A-11R-A220-07 | Cluster_I |
| TCGA-BT-A3PJ-01A-21R-A220-07 | Cluster_III |
| TCGA-BT-A3PK-01A-21R-A220-07 | Cluster_III |
| TCGA-BT-A42C-01A-11R-A23N-07 | Cluster_I |
| TCGA-BT-A42E-01A-11R-A23W-07 | Cluster_III |
| TCGA-BT-A42F-01A-11R-A23W-07 | Cluster_III |
| TCGA-C4-A0EZ-01A-21R-A24X-07 | Cluster_III |
| TCGA-C4-A0F0-01A-12R-A10U-07 | Cluster_III |
| TCGA-C4-A0F1-01A-11R-A034-07 | Cluster_III |
| TCGA-C4-A0F6-01A-11R-A10U-07 | Cluster_I |
| TCGA-C4-A0F7-01A-11R-A084-07 | Cluster_III |
| TCGA-CF-A1HR-01A-11R-A13Y-07 | Cluster_I |
| TCGA-CF-A1HS-01A-11R-A13Y-07 | Cluster_III |
| TCGA-CF-A27C-01A-11R-A16R-07 | Cluster_I |
| TCGA-CF-A3MF-01A-12R-A21D-07 | Cluster_I |
| TCGA-CF-A3MG-01A-11R-A20F-07 | Cluster_I |
| TCGA-CF-A3MH-01A-11R-A20F-07 | Cluster_I |
| TCGA-CF-A3MI-01A-11R-A20F-07 | Cluster_I |
| TCGA-CF-A47S-01A-11R-A23W-07 | Cluster_I |
| TCGA-CF-A47T-01A-11R-A23W-07 | Cluster_I |
| TCGA-CF-A47V-01A-11R-A23W-07 | Cluster_I |
| TCGA-CF-A47W-01A-11R-A23W-07 | Cluster_I |
| TCGA-CF-A47X-01A-31R-A23W-07 | Cluster_I |
| TCGA-CF-A47Y-01A-11R-A23W-07 | Cluster_I |
| TCGA-CF-A5U8-01A-11R-A28M-07 | Cluster_I |
| TCGA-CF-A5UA-01A-11R-A28M-07 | Cluster_I |
| TCGA-CF-A7I0-01A-22R-A352-07 | Cluster_I |
| TCGA-CF-A8HX-01A-11R-A36F-07 | Cluster_I |
| TCGA-CF-A8HY-01A-11R-A36F-07 | Cluster_I |
| TCGA-CF-A9FF-01A-11R-A38B-07 | Cluster_I |
| TCGA-CF-A9FH-01A-11R-A38B-07 | Cluster_I |
| TCGA-CF-A9FL-01A-11R-A38B-07 | Cluster_I |
| TCGA-CF-A9FM-01A-11R-A38B-07 | Cluster_I |
| TCGA-CU-A0YN-01A-21R-A10U-07 | Cluster_III |
| TCGA-CU-A0YO-01A-11R-A10U-07 | Cluster_II |
| TCGA-CU-A0YR-01A-12R-A10U-07 | Cluster_II |
| TCGA-CU-A3KJ-01A-11R-A21D-07 | Cluster_III |
| TCGA-CU-A3QU-01A-11R-A22U-07 | Cluster_I |
| TCGA-CU-A3YL-01A-11R-A22U-07 | Cluster_I |
| TCGA-CU-A5W6-01A-11R-A28M-07 | Cluster_I |
| TCGA-CU-A72E-01A-12R-A33J-07 | Cluster_III |
| TCGA-DK-A1A3-01A-11R-A13Y-07 | Cluster_II |
| TCGA-DK-A1A5-01A-11R-A13Y-07 | Cluster_II |
| TCGA-DK-A1A6-01A-11R-A13Y-07 | Cluster_I |
| TCGA-DK-A1A7-01A-11R-A13Y-07 | Cluster_I |
| TCGA-DK-A1AA-01A-11R-A13Y-07 | Cluster_I |
| TCGA-DK-A1AB-01A-11R-A13Y-07 | Cluster_III |
| TCGA-DK-A1AC-01A-11R-A13Y-07 | Cluster_I |
| TCGA-DK-A1AD-01A-11R-A13Y-07 | Cluster_II |
| TCGA-DK-A1AE-01A-11R-A13Y-07 | Cluster_III |
| TCGA-DK-A1AF-01A-11R-A13Y-07 | Cluster_II |
| TCGA-DK-A1AG-01A-11R-A13Y-07 | Cluster_I |
| TCGA-DK-A2HX-01A-12R-A18C-07 | Cluster_II |
| TCGA-DK-A2I1-01A-11R-A180-07 | Cluster_II |
| TCGA-DK-A2I2-01A-11R-A180-07 | Cluster_III |
| TCGA-DK-A2I4-01A-11R-A21D-07 | Cluster_IV |
| TCGA-DK-A2I6-01A-12R-A18C-07 | Cluster_III |
| TCGA-DK-A3IK-01A-32R-A21D-07 | Cluster_II |
| TCGA-DK-A3IL-01A-11R-A20F-07 | Cluster_II |
| TCGA-DK-A3IM-01A-11R-A20F-07 | Cluster_III |
| TCGA-DK-A3IN-01A-11R-A20F-07 | Cluster_III |
| TCGA-DK-A3IQ-01A-31R-A32Y-07 | Cluster_II |
| TCGA-DK-A3IS-01A-21R-A21D-07 | Cluster_I |
| TCGA-DK-A3IT-01A-31R-A20F-07 | Cluster_II |
| TCGA-DK-A3IU-01A-11R-A20F-07 | Cluster_IV |
| TCGA-DK-A3IV-01A-22R-A21D-07 | Cluster_I |
| TCGA-DK-A3WW-01A-22R-A23N-07 | Cluster_III |
| TCGA-DK-A3WX-01A-22R-A22U-07 | Cluster_III |
| TCGA-DK-A3WY-01A-11R-A22U-07 | Cluster_IV |
| TCGA-DK-A3X1-01A-12R-A22U-07 | Cluster_I |
| TCGA-DK-A3X2-01A-11R-A22U-07 | Cluster_III |
| TCGA-DK-A6AV-01A-12R-A30C-07 | Cluster_I |
| TCGA-DK-A6AW-01A-11R-A30C-07 | Cluster_I |
| TCGA-DK-A6B0-01A-11R-A31N-07 | Cluster_I |
| TCGA-DK-A6B1-01A-12R-A30C-07 | Cluster_I |
| TCGA-DK-A6B2-01A-11R-A30C-07 | Cluster_II |
| TCGA-DK-A6B5-01A-11R-A31N-07 | Cluster_III |
| TCGA-DK-A6B6-01A-11R-A30C-07 | Cluster_I |
| TCGA-DK-AA6L-01A-11R-A39I-07 | Cluster_I |
| TCGA-DK-AA6M-01A-11R-A39I-07 | Cluster_III |
| TCGA-DK-AA6P-01A-11R-A39I-07 | Cluster_I |
| TCGA-DK-AA6Q-01A-11R-A39I-07 | Cluster_III |
| TCGA-DK-AA6R-01A-11R-A42T-07 | Cluster_III |
| TCGA-DK-AA6S-01A-21R-A39I-07 | Cluster_II |
| TCGA-DK-AA6T-01A-11R-A39I-07 | Cluster_IV |
| TCGA-DK-AA6U-01A-11R-A39I-07 | Cluster_I |
| TCGA-DK-AA6W-01A-12R-A39I-07 | Cluster_III |
| TCGA-DK-AA6X-01A-12R-A42T-07 | Cluster_II |
| TCGA-DK-AA71-01A-31R-A39I-07 | Cluster_I |
| TCGA-DK-AA74-01A-11R-A39I-07 | Cluster_IV |
| TCGA-DK-AA75-01A-11R-A39I-07 | Cluster_I |
| TCGA-DK-AA76-01A-11R-A39I-07 | Cluster_I |
| TCGA-DK-AA77-01A-11R-A39I-07 | Cluster_I |
| TCGA-E5-A2PC-01A-11R-A206-07 | Cluster_I |
| TCGA-E5-A4TZ-01A-11R-A31N-07 | Cluster_I |
| TCGA-E5-A4U1-01A-11R-A31N-07 | Cluster_I |
| TCGA-E7-A3X6-01A-12R-A22U-07 | Cluster_I |
| TCGA-E7-A3Y1-01A-11R-A22U-07 | Cluster_I |
| TCGA-E7-A4IJ-01A-31R-A26T-07 | Cluster_I |
| TCGA-E7-A4XJ-01A-11R-A26T-07 | Cluster_I |
| TCGA-E7-A519-01A-11R-A26T-07 | Cluster_I |
| TCGA-E7-A541-01A-11R-A26T-07 | Cluster_I |
| TCGA-E7-A5KE-01A-11R-A28M-07 | Cluster_I |
| TCGA-E7-A5KF-01A-11R-A28M-07 | Cluster_I |
| TCGA-E7-A677-01A-11R-A30C-07 | Cluster_I |
| TCGA-E7-A678-01A-11R-A30C-07 | Cluster_I |
| TCGA-E7-A6MD-01A-41R-A352-07 | Cluster_II |
| TCGA-E7-A6ME-01A-22R-A32O-07 | Cluster_I |
| TCGA-E7-A6MF-01A-12R-A32O-07 | Cluster_I |
| TCGA-E7-A7DU-01A-11R-A32O-07 | Cluster_I |
| TCGA-E7-A7DV-01A-11R-A33J-07 | Cluster_IV |
| TCGA-E7-A7PW-01A-11R-A352-07 | Cluster_I |
| TCGA-E7-A7XN-01A-11R-A352-07 | Cluster_III |
| TCGA-E7-A85H-01A-11R-A352-07 | Cluster_I |
| TCGA-E7-A8O7-01A-11R-A36F-07 | Cluster_I |
| TCGA-E7-A8O8-01A-11R-A36F-07 | Cluster_I |
| TCGA-E7-A97P-01A-11R-A38B-07 | Cluster_III |
| TCGA-E7-A97Q-01A-11R-A38B-07 | Cluster_II |
| TCGA-FD-A3B3-01A-12R-A206-07 | Cluster_III |
| TCGA-FD-A3B4-01A-12R-A206-07 | Cluster_III |
| TCGA-FD-A3B5-01A-11R-A20F-07 | Cluster_III |
| TCGA-FD-A3B6-01A-21R-A20F-07 | Cluster_III |
| TCGA-FD-A3B7-01A-31R-A20F-07 | Cluster_IV |
| TCGA-FD-A3B8-01A-31R-A20F-07 | Cluster_IV |
| TCGA-FD-A3N5-01A-11R-A21D-07 | Cluster_III |
| TCGA-FD-A3N6-01A-11R-A21D-07 | Cluster_III |
| TCGA-FD-A3NA-01A-11R-A21D-07 | Cluster_I |
| TCGA-FD-A3SJ-01A-12R-A22U-07 | Cluster_II |
| TCGA-FD-A3SL-01A-21R-A22U-07 | Cluster_II |
| TCGA-FD-A3SM-01A-11R-A22U-07 | Cluster_II |
| TCGA-FD-A3SN-01A-12R-A22U-07 | Cluster_III |
| TCGA-FD-A3SO-01A-11R-A22U-07 | Cluster_III |
| TCGA-FD-A3SP-01A-31R-A22U-07 | Cluster_IV |
| TCGA-FD-A3SQ-01A-21R-A22U-07 | Cluster_II |
| TCGA-FD-A3SR-01A-11R-A22U-07 | Cluster_II |
| TCGA-FD-A3SS-01A-12R-A22U-07 | Cluster_III |
| TCGA-FD-A43N-01A-11R-A23W-07 | Cluster_I |
| TCGA-FD-A43P-01A-31R-A23W-07 | Cluster_IV |
| TCGA-FD-A43S-01A-21R-A23W-07 | Cluster_II |
| TCGA-FD-A43U-01A-11R-A23W-07 | Cluster_IV |
| TCGA-FD-A43X-01A-11R-A23W-07 | Cluster_I |
| TCGA-FD-A43Y-01A-21R-A26T-07 | Cluster_III |
| TCGA-FD-A5BR-01A-11R-A26T-07 | Cluster_II |
| TCGA-FD-A5BS-01A-21R-A26T-07 | Cluster_IV |
| TCGA-FD-A5BT-01A-11R-A26T-07 | Cluster_IV |
| TCGA-FD-A5BU-01A-31R-A26T-07 | Cluster_III |
| TCGA-FD-A5BV-01A-11R-A26T-07 | Cluster_I |
| TCGA-FD-A5BX-01A-11R-A26T-07 | Cluster_IV |
| TCGA-FD-A5BY-01A-31R-A28M-07 | Cluster_III |
| TCGA-FD-A5BZ-01A-11R-A28M-07 | Cluster_II |
| TCGA-FD-A5C0-01A-11R-A28M-07 | Cluster_II |
| TCGA-FD-A5C1-01A-11R-A28M-07 | Cluster_III |
| TCGA-FD-A62N-01A-11R-A30C-07 | Cluster_IV |
| TCGA-FD-A62O-01A-11R-A30C-07 | Cluster_I |
| TCGA-FD-A62P-01A-32R-A30C-07 | Cluster_III |
| TCGA-FD-A62S-01A-11R-A30C-07 | Cluster_IV |
| TCGA-FD-A6TA-01A-12R-A33J-07 | Cluster_II |
| TCGA-FD-A6TB-01A-12R-A33J-07 | Cluster_IV |
| TCGA-FD-A6TC-01A-21R-A33J-07 | Cluster_II |
| TCGA-FD-A6TD-01A-51R-A33J-07 | Cluster_III |
| TCGA-FD-A6TE-01A-12R-A33J-07 | Cluster_I |
| TCGA-FD-A6TF-01A-52R-A32O-07 | Cluster_II |
| TCGA-FD-A6TG-01A-11R-A32O-07 | Cluster_II |
| TCGA-FD-A6TH-01A-11R-A32O-07 | Cluster_III |
| TCGA-FD-A6TI-01A-11R-A32O-07 | Cluster_II |
| TCGA-FD-A6TK-01A-42R-A33J-07 | Cluster_IV |
| TCGA-FJ-A3Z7-01A-12R-A23N-07 | Cluster_II |
| TCGA-FJ-A3Z9-01A-11R-A26T-07 | Cluster_I |
| TCGA-FJ-A3ZE-01A-11R-A23N-07 | Cluster_I |
| TCGA-FJ-A3ZF-01A-11R-A23N-07 | Cluster_I |
| TCGA-FJ-A871-01A-11R-A352-07 | Cluster_III |
| TCGA-FT-A3EE-01A-11R-A206-07 | Cluster_I |
| TCGA-FT-A61P-01A-11R-A30C-07 | Cluster_IV |
| TCGA-G2-A2EC-01A-11R-A180-07 | Cluster_II |
| TCGA-G2-A2EF-01A-12R-A18C-07 | Cluster_III |
| TCGA-G2-A2EJ-01A-11R-A180-07 | Cluster_III |
| TCGA-G2-A2EK-01A-22R-A18C-07 | Cluster_I |
| TCGA-G2-A2EL-01A-12R-A18C-07 | Cluster_III |
| TCGA-G2-A2EO-01A-11R-A180-07 | Cluster_II |
| TCGA-G2-A2ES-01A-11R-A180-07 | Cluster_III |
| TCGA-G2-A3IB-01A-11R-A20F-07 | Cluster_III |
| TCGA-G2-A3IE-01A-11R-A20F-07 | Cluster_I |
| TCGA-G2-A3VY-01A-11R-A22U-07 | Cluster_I |
| TCGA-G2-AA3B-01A-11R-A39I-07 | Cluster_I |
| TCGA-G2-AA3C-01A-21R-A39I-07 | Cluster_IV |
| TCGA-G2-AA3D-01A-11R-A39I-07 | Cluster_I |
| TCGA-G2-AA3F-01A-12R-A42T-07 | Cluster_I |
| TCGA-GC-A3BM-01A-11R-A22U-07 | Cluster_I |
| TCGA-GC-A3I6-01A-11R-A20F-07 | Cluster_III |
| TCGA-GC-A3OO-01A-11R-A22U-07 | Cluster_II |
| TCGA-GC-A3RB-01A-12R-A220-07 | Cluster_I |
| TCGA-GC-A3RC-01A-11R-A22U-07 | Cluster_III |
| TCGA-GC-A3RD-01A-12R-A22U-07 | Cluster_I |
| TCGA-GC-A3WC-01A-31R-A22U-07 | Cluster_III |
| TCGA-GC-A3YS-01A-11R-A23N-07 | Cluster_III |
| TCGA-GC-A4ZW-01A-11R-A26T-07 | Cluster_I |
| TCGA-GC-A6I1-01A-12R-A31N-07 | Cluster_IV |
| TCGA-GC-A6I3-01A-11R-A31N-07 | Cluster_II |
| TCGA-GD-A2C5-01A-12R-A180-07 | Cluster_II |
| TCGA-GD-A3OP-01A-21R-A220-07 | Cluster_II |
| TCGA-GD-A3OQ-01A-32R-A220-07 | Cluster_III |
| TCGA-GD-A3OS-01A-12R-A220-07 | Cluster_IV |
| TCGA-GD-A6C6-01A-21R-A31N-07 | Cluster_I |
| TCGA-GD-A76B-01A-11R-A32O-07 | Cluster_I |
| TCGA-GU-A42P-01A-11R-A23W-07 | Cluster_I |
| TCGA-GU-A42Q-01A-11R-A23W-07 | Cluster_III |
| TCGA-GU-A42R-01A-11R-A23N-07 | Cluster_I |
| TCGA-GU-A762-01A-11R-A33J-07 | Cluster_IV |
| TCGA-GU-A763-01A-11R-A32O-07 | Cluster_I |
| TCGA-GU-A764-01A-11R-A352-07 | Cluster_III |
| TCGA-GU-A766-01A-11R-A32O-07 | Cluster_IV |
| TCGA-GU-A767-01A-11R-A32O-07 | Cluster_II |
| TCGA-GU-AATO-01A-11R-A39I-07 | Cluster_II |
| TCGA-GU-AATP-01A-11R-A39I-07 | Cluster_II |
| TCGA-GU-AATQ-01A-11R-A39I-07 | Cluster_III |
| TCGA-GV-A3JV-01A-11R-A220-07 | Cluster_I |
| TCGA-GV-A3JW-01A-11R-A20F-07 | Cluster_I |
| TCGA-GV-A3JX-01A-11R-A20F-07 | Cluster_I |
| TCGA-GV-A3JZ-01A-11R-A21D-07 | Cluster_I |
| TCGA-GV-A3QF-01A-31R-A22U-07 | Cluster_I |
| TCGA-GV-A3QG-01A-11R-A220-07 | Cluster_IV |
| TCGA-GV-A3QH-01A-11R-A220-07 | Cluster_I |
| TCGA-GV-A3QI-01A-11R-A220-07 | Cluster_I |
| TCGA-GV-A3QK-01B-11R-A23N-07 | Cluster_I |
| TCGA-GV-A40E-01A-12R-A23N-07 | Cluster_III |
| TCGA-GV-A40G-01A-11R-A23N-07 | Cluster_I |
| TCGA-GV-A6ZA-01A-12R-A33J-07 | Cluster_I |
| TCGA-H4-A2HO-01A-11R-A180-07 | Cluster_I |
| TCGA-H4-A2HQ-01A-11R-A180-07 | Cluster_I |
| TCGA-HQ-A2OE-01A-11R-A206-07 | Cluster_I |
| TCGA-HQ-A2OF-01A-11R-A26T-07 | Cluster_I |
| TCGA-HQ-A5ND-01A-11R-A26T-07 | Cluster_III |
| TCGA-HQ-A5NE-01A-12R-A28M-07 | Cluster_III |
| TCGA-K4-A3WS-01A-11R-A22U-07 | Cluster_II |
| TCGA-K4-A3WU-01B-11R-A23N-07 | Cluster_II |
| TCGA-K4-A3WV-01A-11R-A22U-07 | Cluster_III |
| TCGA-K4-A4AB-01B-12R-A28M-07 | Cluster_II |
| TCGA-K4-A4AC-01A-21R-A26T-07 | Cluster_III |
| TCGA-K4-A54R-01A-11R-A26T-07 | Cluster_II |
| TCGA-K4-A5RH-01A-11R-A30C-07 | Cluster_IV |
| TCGA-K4-A5RI-01A-11R-A28M-07 | Cluster_III |
| TCGA-K4-A5RJ-01A-11R-A28M-07 | Cluster_IV |
| TCGA-K4-A6FZ-01A-11R-A31N-07 | Cluster_III |
| TCGA-K4-A6MB-01A-11R-A31N-07 | Cluster_II |
| TCGA-K4-A83P-01A-11R-A352-07 | Cluster_IV |
| TCGA-K4-AAQO-01A-11R-A38B-07 | Cluster_II |
| TCGA-KQ-A41N-01A-11R-A33J-07 | Cluster_I |
| TCGA-KQ-A41O-01A-12R-A352-07 | Cluster_I |
| TCGA-KQ-A41P-01A-12R-A33J-07 | Cluster_II |
| TCGA-KQ-A41Q-01A-11R-A33J-07 | Cluster_I |
| TCGA-KQ-A41R-01A-21R-A352-07 | Cluster_I |
| TCGA-KQ-A41S-01A-12R-A33J-07 | Cluster_II |
| TCGA-LC-A66R-01A-41R-A30C-07 | Cluster_III |
| TCGA-LT-A5Z6-01A-11R-A28M-07 | Cluster_I |
| TCGA-LT-A8JT-01A-11R-A36F-07 | Cluster_I |
| TCGA-MV-A51V-01A-11R-A26T-07 | Cluster_I |
| TCGA-PQ-A6FI-01A-11R-A31N-07 | Cluster_III |
| TCGA-PQ-A6FN-01A-11R-A31N-07 | Cluster_III |
| TCGA-R3-A69X-01A-22R-A30C-07 | Cluster_II |
| TCGA-S5-A6DX-01A-11R-A31N-07 | Cluster_II |
| TCGA-S5-AA26-01A-11R-A38B-07 | Cluster_I |
| TCGA-SY-A9G0-01A-12R-A38B-07 | Cluster_II |
| TCGA-SY-A9G5-01A-11R-A38B-07 | Cluster_II |
| TCGA-UY-A78K-01A-11R-A33J-07 | Cluster_II |
| TCGA-UY-A78L-01A-12R-A33J-07 | Cluster_III |
| TCGA-UY-A78M-01A-21R-A352-07 | Cluster_I |
| TCGA-UY-A78N-01A-12R-A33J-07 | Cluster_I |
| TCGA-UY-A78O-01A-12R-A33J-07 | Cluster_I |
| TCGA-UY-A78P-01A-12R-A36F-07 | Cluster_III |
| TCGA-UY-A8OB-01A-12R-A42T-07 | Cluster_III |
| TCGA-UY-A8OC-01A-11R-A36F-07 | Cluster_III |
| TCGA-UY-A8OD-01A-11R-A36F-07 | Cluster_II |
| TCGA-UY-A9PA-01A-11R-A38B-07 | Cluster_I |
| TCGA-UY-A9PB-01A-11R-A38B-07 | Cluster_IV |
| TCGA-UY-A9PD-01A-11R-A38B-07 | Cluster_I |
| TCGA-UY-A9PE-01A-11R-A38B-07 | Cluster_I |
| TCGA-UY-A9PF-01A-11R-A38B-07 | Cluster_I |
| TCGA-UY-A9PH-01A-11R-A38B-07 | Cluster_II |
| TCGA-XF-A8HB-01A-11R-A36F-07 | Cluster_I |
| TCGA-XF-A8HC-01A-11R-A36F-07 | Cluster_I |
| TCGA-XF-A8HD-01A-11R-A36F-07 | Cluster_III |
| TCGA-XF-A8HE-01A-11R-A36F-07 | Cluster_III |
| TCGA-XF-A8HF-01A-11R-A36F-07 | Cluster_II |
| TCGA-XF-A8HG-01A-11R-A36F-07 | Cluster_I |
| TCGA-XF-A8HH-01A-11R-A38B-07 | Cluster_II |
| TCGA-XF-A8HI-01A-11R-A38B-07 | Cluster_I |
| TCGA-XF-A9SH-01A-11R-A39I-07 | Cluster_I |
| TCGA-XF-A9SI-01A-11R-A39I-07 | Cluster_IV |
| TCGA-XF-A9SJ-01A-11R-A39I-07 | Cluster_II |
| TCGA-XF-A9SK-01A-11R-A42T-07 | Cluster_II |
| TCGA-XF-A9SL-01A-11R-A39I-07 | Cluster_II |
| TCGA-XF-A9SM-01A-11R-A42T-07 | Cluster_IV |
| TCGA-XF-A9SP-01A-11R-A39I-07 | Cluster_II |
| TCGA-XF-A9ST-01A-11R-A42T-07 | Cluster_III |
| TCGA-XF-A9SU-01A-31R-A39I-07 | Cluster_II |
| TCGA-XF-A9SV-01A-21R-A42T-07 | Cluster_II |
| TCGA-XF-A9SW-01A-11R-A42T-07 | Cluster_II |
| TCGA-XF-A9SX-01A-21R-A39I-07 | Cluster_IV |
| TCGA-XF-A9SY-01A-21R-A42T-07 | Cluster_IV |
| TCGA-XF-A9SZ-01A-11R-A39I-07 | Cluster_II |
| TCGA-XF-A9T0-01A-11R-A39I-07 | Cluster_I |
| TCGA-XF-A9T2-01A-11R-A42T-07 | Cluster_III |
| TCGA-XF-A9T3-01A-11R-A42T-07 | Cluster_II |
| TCGA-XF-A9T4-01A-11R-A39I-07 | Cluster_III |
| TCGA-XF-A9T5-01A-11R-A42T-07 | Cluster_IV |
| TCGA-XF-A9T6-01A-11R-A42T-07 | Cluster_III |
| TCGA-XF-A9T8-01A-11R-A39I-07 | Cluster_III |
| TCGA-XF-AAME-01A-12R-A42T-07 | Cluster_IV |
| TCGA-XF-AAMG-01A-11R-A42T-07 | Cluster_III |
| TCGA-XF-AAMH-01A-11R-A42T-07 | Cluster_II |
| TCGA-XF-AAMJ-01A-11R-A42T-07 | Cluster_II |
| TCGA-XF-AAML-01A-11R-A42T-07 | Cluster_I |
| TCGA-XF-AAMQ-01A-11R-A42T-07 | Cluster_I |
| TCGA-XF-AAMR-01A-31R-A42T-07 | Cluster_II |
| TCGA-XF-AAMT-01A-11R-A42T-07 | Cluster_III |
| TCGA-XF-AAMW-01A-11R-A42T-07 | Cluster_III |
| TCGA-XF-AAMX-01A-11R-A42T-07 | Cluster_I |
| TCGA-XF-AAMY-01A-11R-A42T-07 | Cluster_II |
| TCGA-XF-AAMZ-01A-11R-A42T-07 | Cluster_I |
| TCGA-XF-AAN0-01A-11R-A42T-07 | Cluster_II |
| TCGA-XF-AAN1-01A-31R-A42T-07 | Cluster_I |
| TCGA-XF-AAN2-01A-11R-A42T-07 | Cluster_III |
| TCGA-XF-AAN3-01A-11R-A42T-07 | Cluster_II |
| TCGA-XF-AAN4-01A-11R-A42T-07 | Cluster_II |
| TCGA-XF-AAN5-01A-11R-A42T-07 | Cluster_III |
| TCGA-XF-AAN7-01A-11R-A42T-07 | Cluster_IV |
| TCGA-XF-AAN8-01A-11R-A42T-07 | Cluster_IV |
| TCGA-YC-A89H-01A-11R-A36F-07 | Cluster_I |
| TCGA-YC-A8S6-01A-31R-A38B-07 | Cluster_II |
| TCGA-YC-A9TC-01A-22R-A39I-07 | Cluster_III |
| TCGA-YF-AA3L-01A-11R-A38B-07 | Cluster_I |
| TCGA-YF-AA3M-01A-11R-A42T-07 | Cluster_III |
| TCGA-ZF-A9R0-01A-11R-A38B-07 | Cluster_II |
| TCGA-ZF-A9R1-01A-11R-A39I-07 | Cluster_I |
| TCGA-ZF-A9R2-01A-11R-A39I-07 | Cluster_I |
| TCGA-ZF-A9R3-01A-11R-A38B-07 | Cluster_I |
| TCGA-ZF-A9R4-01A-11R-A38B-07 | Cluster_I |
| TCGA-ZF-A9R5-01A-12R-A42T-07 | Cluster_I |
| TCGA-ZF-A9R7-01A-11R-A38B-07 | Cluster_I |
| TCGA-ZF-A9R9-01A-11R-A38B-07 | Cluster_II |
| TCGA-ZF-A9RC-01A-11R-A38B-07 | Cluster_II |
| TCGA-ZF-A9RD-01A-11R-A42T-07 | Cluster_III |
| TCGA-ZF-A9RE-01A-11R-A38B-07 | Cluster_III |
| TCGA-ZF-A9RF-01A-11R-A38B-07 | Cluster_IV |
| TCGA-ZF-A9RL-01A-11R-A38B-07 | Cluster_I |
| TCGA-ZF-A9RM-01A-11R-A38B-07 | Cluster_I |
| TCGA-ZF-A9RN-01A-11R-A42T-07 | Cluster_III |
| TCGA-ZF-AA4N-01A-11R-A38B-07 | Cluster_III |
| TCGA-ZF-AA4R-01A-11R-A38B-07 | Cluster_II |
| TCGA-ZF-AA4T-01A-11R-A38B-07 | Cluster_I |
| TCGA-ZF-AA4U-01A-11R-A38B-07 | Cluster_I |
| TCGA-ZF-AA4V-01A-11R-A38B-07 | Cluster_III |
| TCGA-ZF-AA4W-01A-12R-A38B-07 | Cluster_III |
| TCGA-ZF-AA4X-01A-11R-A38B-07 | Cluster_I |
| TCGA-ZF-AA51-01A-21R-A39I-07 | Cluster_II |
| TCGA-ZF-AA52-01A-12R-A39I-07 | Cluster_II |
| TCGA-ZF-AA53-01A-11R-A39I-07 | Cluster_III |
| TCGA-ZF-AA54-01A-11R-A39I-07 | Cluster_IV |
| TCGA-ZF-AA56-01A-31R-A39I-07 | Cluster_III |
| TCGA-ZF-AA58-01A-12R-A42T-07 | Cluster_IV |
| TCGA-ZF-AA5H-01A-11R-A39I-07 | Cluster_III |
| TCGA-ZF-AA5N-01A-11R-A42T-07 | Cluster_I |
| TCGA-ZF-AA5P-01A-11R-A39I-07 | Cluster_II |

**Supp Figure Legends**

**Supp Figure 1**

*Micrographs of ERBB2 FISH and IHC in bladder cancer samples*

A) FISH images of tumors with normal and amplified ERBB2 status in the upper and lower panel, respectively. Scale bar represents 10 µm.

B) Representative IHC images from the NAC TMA. Her2 Score 0 and 3 in the left and right panel, respectively. Scale bar represents 200 µm.

**Supp Figure 2**

*Data of ERBB2 methylation-, gene status and relation to expression*

A) ERBB2 mRNA expression was significantly associated with high protein expression in our NAC cohort (lr, rl: Score 0-1 in IHC).

B) Correlation of ERBB2 mRNA (x-axis) and protein (y-axis) expression in the TCGA cohort (Pearson).

C) ERBB2 methylation rate (y-axis) compared with ERBB2 CNV (x-axis) in the TCGA cohort. Amplified cases had the lowest rate of ERBB2 methylation. (lr, rl: hemizygous deletion).

D) Cases of the TCGA cohort with ERBB2 amplification only. Amplified cases with a low mRNA expression had the highest rate of ERBB2 methylation (Pearson).

E) ERBB2 copy number as determined by exome sequencing compared to the ERBB2 gene amplification status determined by FISH. Amplified cases had highest copy number by exome sequencing (lr, rl: FISH status negative).

F) Boxplots showing the same comparison as (C), but using sequencing copy number thresholds as the baseline (lr, rl: CNV hemizygous deletion).

G) Bardiagram of FISH status and CNV by exome sequencing. Both methods supported each other, with amplified samples in FISH also being most frequently amplified in CNV. However, none of the cases with hemizygous deletion was equivocal or amplified in FISH (Fisher’s test).

**Supp Figure 3**

*Isolated copy number alterations of ERBB2 in the TCGA cohort*

Copy number plots of the three cases in the TCGA cohort with isolated ERBB2 amplification. Of 64 genes (Supp Table 2) that are known to show alterations in MIBC only ERBB2 was amplified in these three cases. The genes are in alphabetic order from left to right as in Supp Table 2.

**Supp Figure 4**

*Association of Her2 alterations with tumor molecular subtypes*

(A) Barplot showing that amplification in ERBB2 was more common in luminal tumors (cluster I and II). This trend was only significant in the TCGA cohort that contains expanded numbers of tumors (Figure 4A, Fisher).

The lower panels demonstrate that samples classified as TCGA cluster I have the highest ERBB2 mRNA (B) and protein (C) expression (lr, rl: Cluster I).

**Supp Figure 5**

*Association of Her2 alteration with overall survival*

Kaplan-Meier plots showing patient outcomes in the NAC cohort (A, C, E) and in the TCGA cohort (B, D, F), stratified by Her2 alterations (A, B = gene amplification; C, D = mRNA expression; E, F = protein expression). Note that Her2 alteration status did not demonstrate association with overall survival either in the NAC and the TCGA cohort.

**
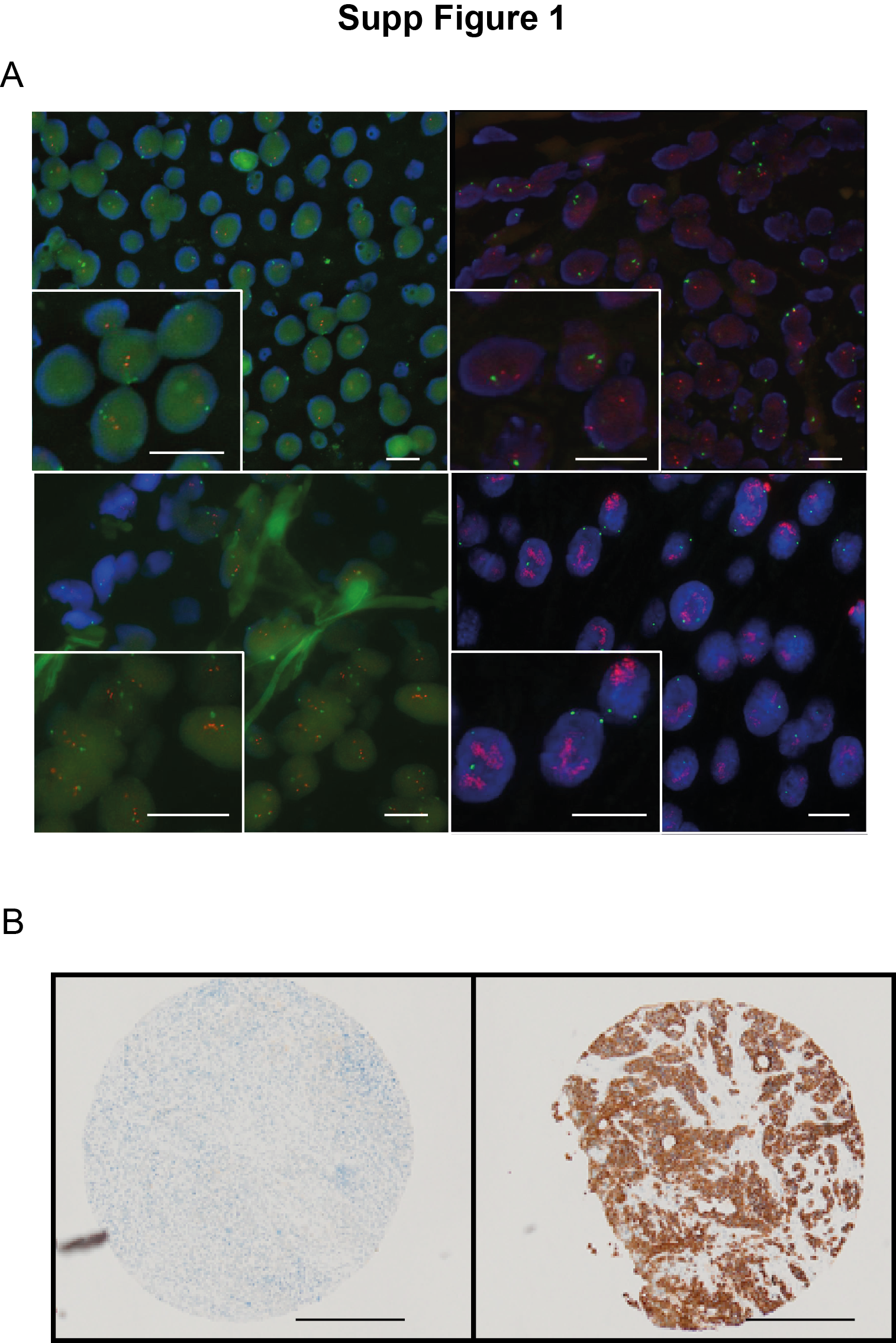
**

**
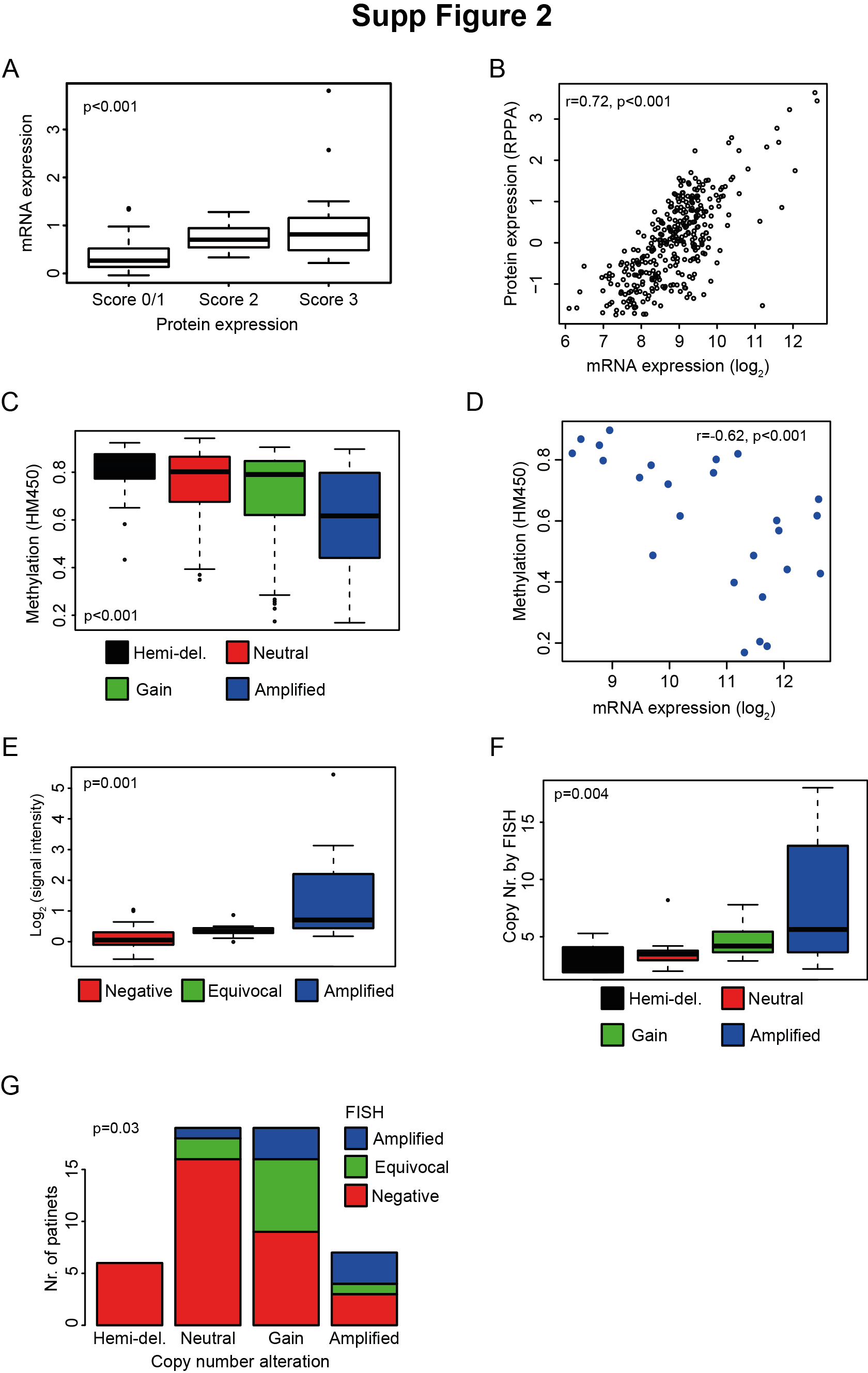
**

**
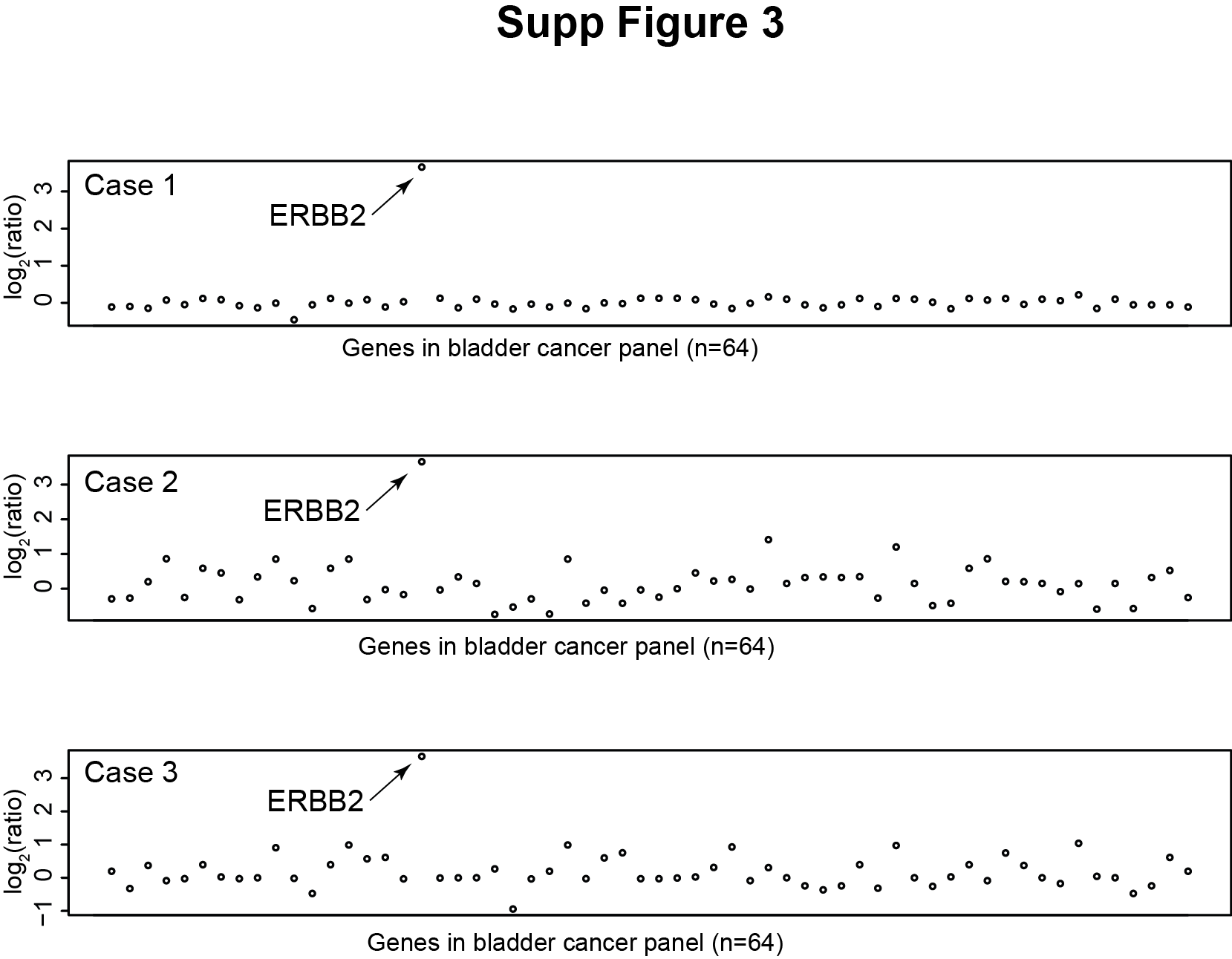
**

**
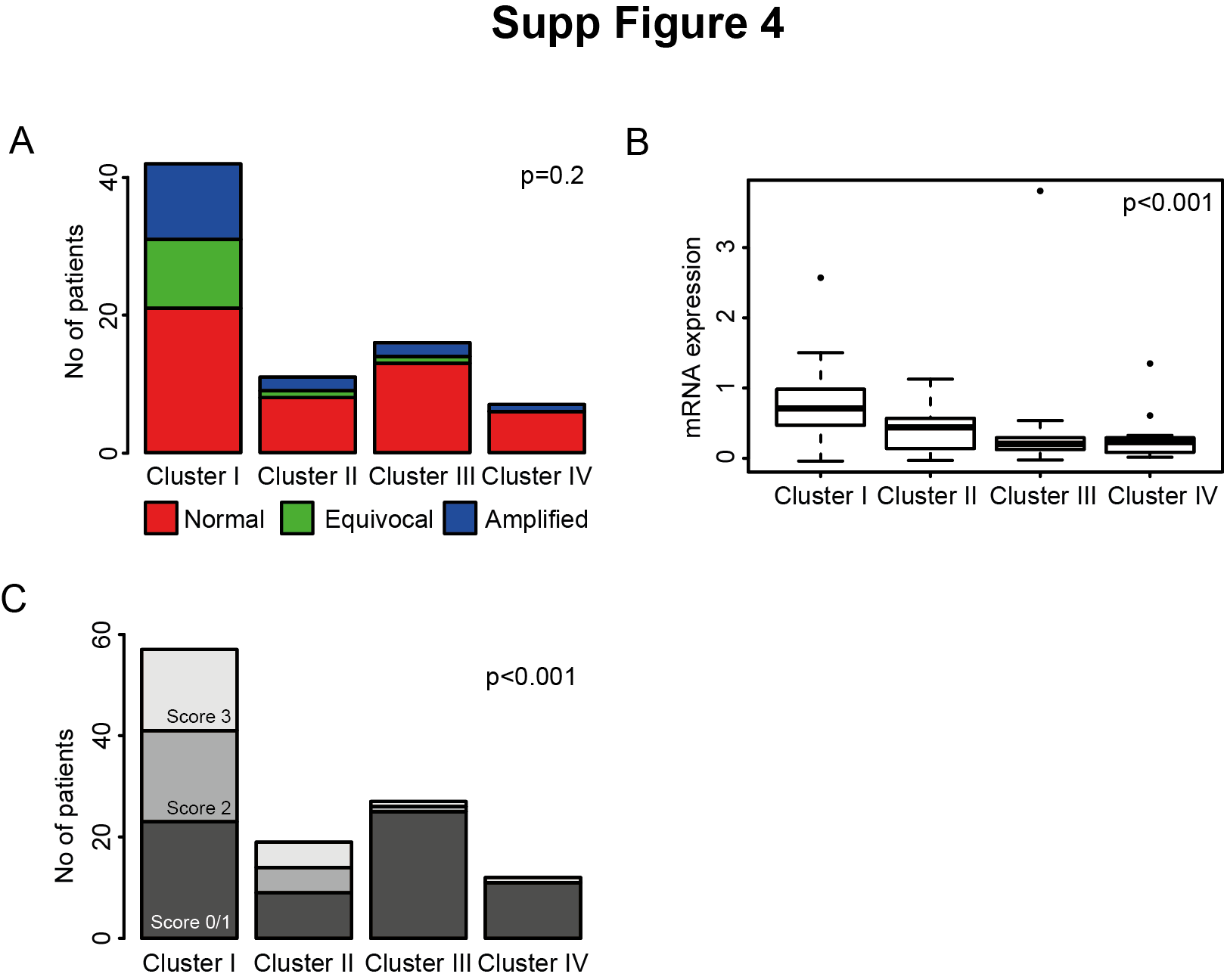
**

**
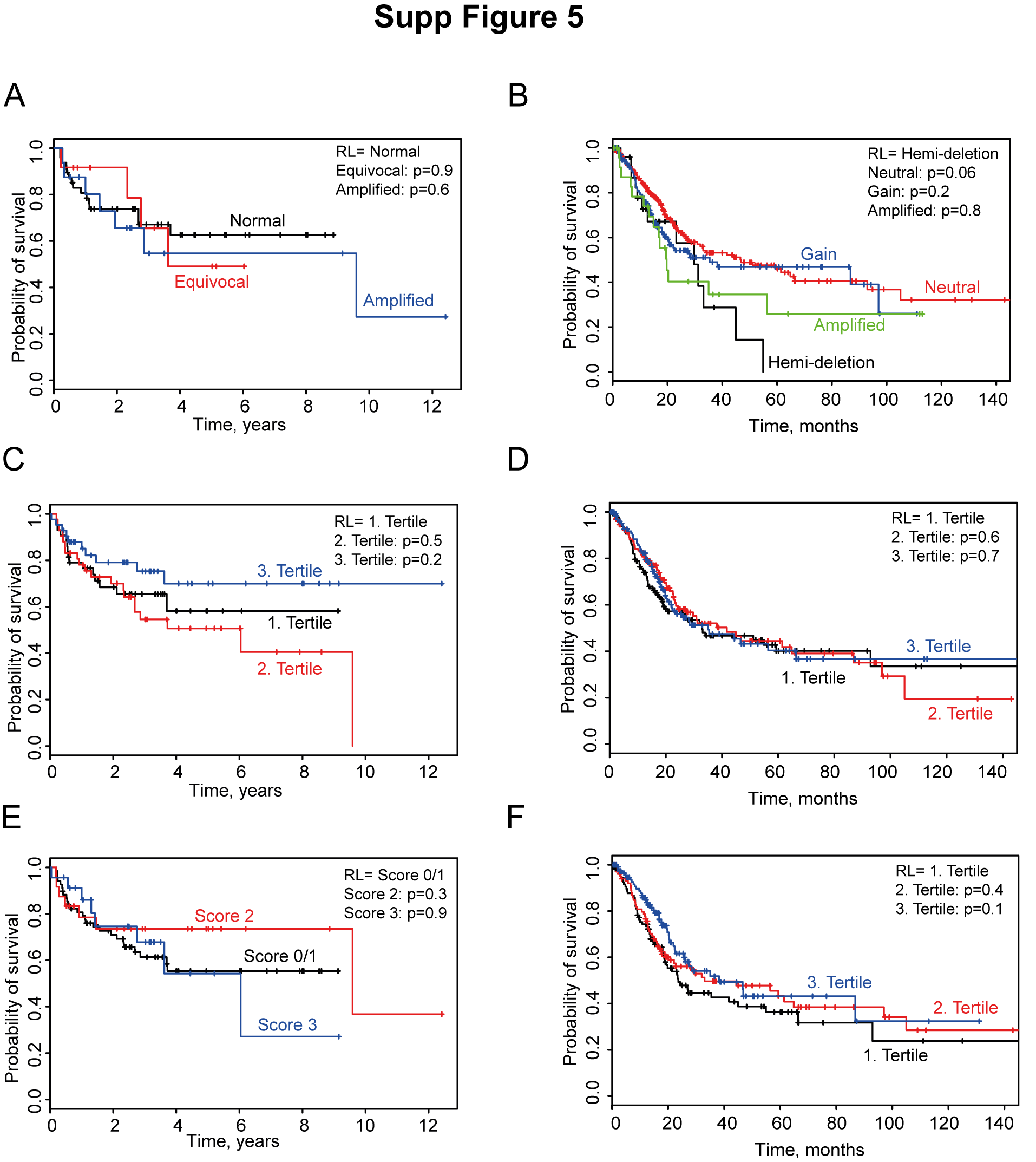
**
